# On a greedy approach for genome scaffolding

**DOI:** 10.1186/s13015-022-00223-x

**Published:** 2022-10-29

**Authors:** Tom Davot, Annie Chateau, Rohan Fossé, Rodolphe Giroudeau, Mathias Weller

**Affiliations:** 1 LIRMM, Univ. Montpellier, Montpellier, France; 2 IBC, Montpellier, France; 3 LaBRI, University of Bordeaux, Bordeaux, France; 4 CNRS, LIGM, Université Gustave Eiffel, Champs-s/-Marne, France

**Keywords:** Genome scaffolding, Complexity, Approximation, Dynamic programming, Poly-APX-hardness

## Abstract

**Background:**

Scaffolding is a bioinformatics problem aimed at completing the contig assembly process by determining the relative position and orientation of these contigs. It can be seen as a paths and cycles cover problem of a particular graph called the “scaffold graph”.

**Results:**

We provide some NP-hardness and inapproximability results on this problem. We also adapt a greedy approximation algorithm on complete graphs so that it works on a special class aiming to be close to real instances. The described algorithm is the first polynomial-time approximation algorithm designed for this problem on non-complete graphs.

**Conclusion:**

Tests on a set of simulated instances show that our algorithm provides better results than the version on complete graphs.

## Background

### Motivation

In this paper, we focus on a bioinformatic problem occurring in the production of genomes. Genomes are usually obtained by sequencing. Sequencing produces an important amount of small sequences of nucleotides called *reads*. Herein, the lengths range from hundreds to tens of thousands of characters, depending on the sequencing technology. As a rule of thumb, shorter reads, produced for example by second generation sequencing (Illumina) have a higher quality (contain less read-errors) than long reads produced by third generation sequencing technologies (PacBio or Oxford Nanopore) [1]. The *assembly* process exploits overlaps between reads to reconstruct the targeted sequence. However, this is complicated by repeated parts in real-world genomes. Assembly algorithms cannot uniquely infer the original genome if it contains such repeated regions (the longer the repeated region with respect to the read length, the harder it is to infer the original genome). To avoid misassembly, such algorithms reconstruct only parts of the genome which is then returned as set of “contiguous regions” (or *contigs*). A thus fragmented genome is not ideal for further processing, and one would like to have as few contigs as possible while avoiding misassembly. A way to approach this are hybrid strategies using both long and short reads [2]. However, many genomes comprising current databases have been assembled before the development of third generation sequencing, preventing such hybrid strategies. One way to reduce the fragmentation of genomes in these databases while avoiding costly re-sequencing, is the exploitation of “meta-information” about the available reads.

### Genome scaffolding

In second generation sequencing, short reads come in pairs, indicating that a fragment of the DNA molecule exists whose ends correspond to the two reads of a pair. In particular, the total length of said fragment is known approximately. This pairing information can be used to infer the order (and orientation) of the given contigs on the chromosome, thus completing the genome (modulo possible gaps between the contigs). The mathematical problem modeling this inference, called scaffolding, is made complicated by possible inconsistencies in the pairing information. See [3] for a recent overview of models, variants, and methods in this context.

The problem we study here is an optimization problem in a special graph called *scaffold graph*. The present formulation use both pairing information and some genomic structural constraints, like a fixed number of linear and circular chromosomes. In [4], we presented preliminary results about the complexity of this problem and a first polynomial-time approximation on complete graphs. Those results were extended and completed by another polynomial-time algorithm [5] and by a randomized approach [6]. We also explored exact algorithms [7], and studied some sparse special cases of scaffold graphs [8]. The contribution of the present paper is a continuation of published works [9, 10], where special classes of graphs have been studied (from sparse to very dense). Since real instances are usually sparse but contain some dense regions, due to abundance of repeats [11], we are interested in graphs built from cliques that are separated by bridges (i.e. edges whose removal disconnects the graph). The main contribution is the extension of the approximation algorithm on complete graphs of Chateau and Giroudeau [5] to a particular class called “connected cluster graph ”. Ultimately, the objective is to adapt the algorithm to sparse classes of graphs. To keep the approximation algorithm in polynomial time, one condition is that the decision problem of the scaffolding must be solvable in polynomial time. We propose a negative result, (i.e. it is $\mathcal{NP}\mathcal{}$-complete) for a particular sparse graph class. Finally, since the presented approximation has a polynomial approximation ratio in some particular cases, we show that the scaffolding problem can not be approximed with a ratio better than a polynomial function in such cases.

### Organization of the paper

The next section is devoted to notations and the description of the scaffold problem. In "Computational hardness" section, we show a $\mathcal{NP}\mathcal{}$-hardness results for sparse scaffolding graphs. In "Non-approximability" section, we address inapproximability. "Feasibility function for connected cluster graphs" section is devoted to a greedy algorithm for a special class of graph called connected cluster graph. Finally, we provide experimental results for the greedy algorithm.

## Notation and problem description

### Graph definitions

For a graph *G*, we denote by *V*(*G*) and *E*(*G*) the set of vertices and edges of *G*, respectively. Let *u* be a vertex of *G*, the *degree* *d*(*u*) of *u* is the number of edges incident with *u*. The *girth* *g*(*G*) of *G* is the length of the smallest cycle of *G*. A graph is *bipartite* if its vertices can be partitioned into two sets of non-adjacent vertices. A graph is *planar* if it can be drawn in the two-dimensional plane without crossing edges.

A matching $M^*\subseteq E(G)$ of *G* is a set of non-adjacent edges. $M^*$ is called *perfect* if it touches all vertices of *G*. For a vertex *u*, we let $M^*(u)$ denote the unique vertex *v* (if it exists) such that $uv \in M^*$. In a scaffold graph, vertices represent extremities of contigs. Given a matching $M^*$, the matching edges represent contigs and edges outside the matching represent possible contiguity relationship between contigs. The confidence that two contigs (more precisely, contig-extremities) occur consecutively in the genomic sequence is represented by a weight on edges outside the matching. An *alternating path* (resp. *alternating cycle*) is a path (resp. cycle) such that its edges alternatingly belong to $M^*$ or not. The extremal edges of an alternating path must be in $M^*$.

A *clique* of *G* is a set of vertices such that all vertices are adjacent. A *bridge* (resp. *cut vertex*) of *G* is an edge (resp. vertex) such that its deletion increases by one the number of connected components of *G*. In "Feasibility function for connected cluster graphs" section, we study a particular class of graph called connected cluster graph, defined as follows.

#### Definition 1

A connected cluster graph *G* is a graph that admits a decomposition of its edges $E(G)=E'\cup B$ such that the subgraph induced by $E'$ is a disjoint union of cliques and each edge $e \in B$ is a bridge of *G*.

**Fig. 1:**
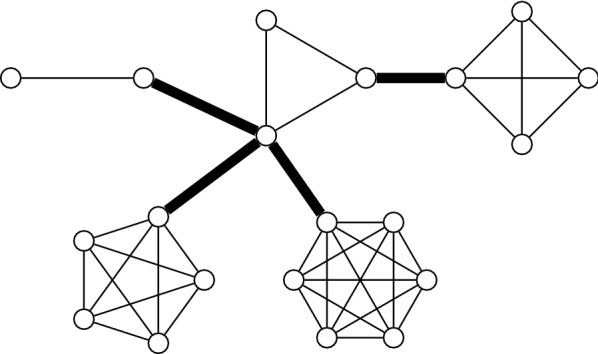
Example of a connected cluster graph. The bridge edges are bold

An example of a connected cluster graph is given in Fig. 1.

### Scaffolding problem

A *scaffold graph* $(G^*,M^*,\omega )$ is a simple, loopless graph $G^*$ with a perfect matching $M^*$ and a weight function $\omega$ on the non-matching edges. The matching $M^*$ represents the set of contigs and for an edge *uv*, $\omega (uv)$ indicates the confidence that the contig extremity *v* follows the contig extremity *u* in the genomic sequence.^1^ The *alternating girth* of a scaffold graph denoted by $g^*(G^*)$ is the number of matching edges in the smallest alternating cycle of $(G^*,M^*,\omega )$. In this paper, we study a decision and optimization version of scaffolding, defined as follows.
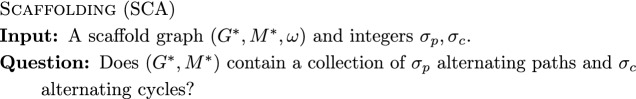

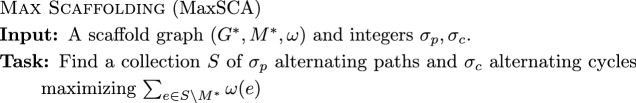


The two integers $\sigma _p$ and $\sigma _c$ are used to model restrictions on the sought genomic structure by representing the number of linear and circular chromosomes, respectively. 
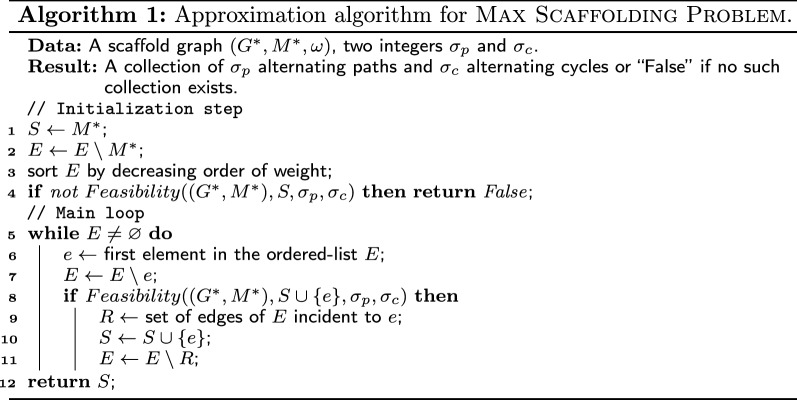
 Let *S* be a collection of *p* alternating paths and *c* alternating cycles. We call the number $p+c$ the *cardinality* of *S* and, we let $\sigma _p(S):=p$ and $\sigma _c(S):=c$.

## Greedy algorithm

The main contribution of this paper is an extension of a known polynomial-time 3-approximation [5] to connected cluster graphs. Whereas the original algorithm was developed to work in complete graphs, it can be adapted for the general case, as shown in Algorithm 1. The idea of this greedy algorithm is to consider each non-matching edge in decreasing order of weight and add it into a partial solution, if possible. The key instruction is the feasibility function: given a partial solution *S* and an edge *e*, this function indicates whether $S\cup e$ can still be extended into a collection of $\sigma _c$ alternating cycles and $\sigma _p$ alternating paths in $(G^*,M^*)$.

### Proposition 1

Let *f* be a feasibility function with time complexity $\mathcal {O}(t)$. Algorithm 1 gives an approximate solution for max scaffolding (if it exists) in $\mathcal {O}(|E(G^*)| \cdot (t+\log |E(G^*)|))$.

The solution *S* given in the input of the feasibility function is called *initiating solution*. In general, since scaffolding is $\mathcal{NP}\mathcal{}$-complete, feasibility cannot be decided in polynomial-time, even if $S=\varnothing$ (unless $\mathcal {P}$ =$\mathcal{NP}\mathcal{}$). Thus, we focus on restricted classes of graphs. In [5], a constant-time feasibility function was developed for complete graphs, leading to the following result.

### Theorem 1

([5]) In complete graphs, Algorithm 1 gives a solution with an approximation factor of 3.

**Fig. 2:**
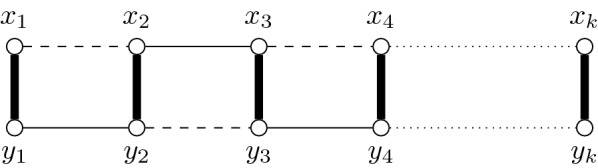
Unbounded ratio of Algorithm 1 in the general case. Let $(G^*,M^*,\omega )$ be a $2 \times k$ grid where the perfect matching (bold edges) corresponds to the edges between the two rows. Let $(x_1,\dots ,x_k)$ and $(y_1,\dots ,y_k)$ be the vertices of the first and second row, respectively. We are looking for a solution of max scaffolding with $\sigma _c =0$ and $\sigma _p=1$. If the algorithm chooses first the edge $x_1x_2$, then the only feasible solution is $S = \{x_{\ell }x_{\ell } \mid \ell \mod 2 = 1 \} \cup \{ y_{\ell }y_{\ell +1} \mid \ell \mod 2 = 0 \}$ (dashed edges). Suppose that an optimal solution is $S_{opt}=E(G^*)\setminus (M^*\cup S)$ (solid edges). If all edges of $S_{opt}$ and $x_1x_2$ are valued by one and all edges of $S \setminus \{x_1x_2\}$ are valued by zero, then we have $(k-1)\cdot \omega (S) = \omega (S_{opt})$ which leads to an unbounded ratio

In "Feasibility function for connected cluster graphs" section, we develop a feasibility function for connected cluster graphs and show that Algorithm 1 gives a 5-approximate solution in this case. Notice that, on graph classes containing the $2\times k$ grids, the worst-case approximation factor of the greedy algorithm cannot be better than polynomial, even if a polynomial-time feasibility function exists (see Fig. 2).

We conclude this section with a note on real-world instances, which are too sparse to fall into our considered class. However, we can transform them by adding some non-matching edges with weight zero. This technique was used to run the feasibility function for complete graphs on simulated instances [5] and the computed solution was close to the optimal. One of the reasons we develop a feasibility function for connected cluster graphs is that we conjecture that using a feasibility function for a graph class that is closer to the original instance (edge-deletion distance from the class) provides better approximation in practice, even though the theoretical approximation factor of the algorithm becomes worse. We test this hypothesis in "Experimental results" section.

## Computational hardness

Like said in the previous section, when using the greedy algorithm on a real instance, we must complete the original instance by adding non-matching edges with weight zero. To minimize the number of added edges, the solution is to adapt the greedy algorithm to a sparse class of graphs. In order to do that, scaffolding must be solvable in polynomial time in this particular class since otherwise, the feasibility function can not be run in polynomial time. In this section, we show that scaffolding is $\mathcal{NP}\mathcal{}$-hard for the particular class of graphs where $|M^*|=2\sigma _c + \sigma _p$. That is, we show that the greedy algorithm can not be executed in polynomial time in this special case. In such instance, any feasible solution *S* contains only alternating paths of length one and alternating cycles of length four (i.e. the smallest possible elements). While scaffolding is polynomial in this case [5], a natural extension would be to consider slightly longer alternating paths and alternating cycles. Unfortunately however, it turns out that deciding whether $(G^*,M^*)$ contains a collection with alternating paths of length one and alternating cycles of length six is already $\mathcal{NP}\mathcal{}$-complete. In order to show this, we focus on the value of the alternating girth of the scaffold graph. Indeed, in a solution of scaffolding with $g^*(G^*) \cdot \sigma _c + \sigma _p$ edges, each alternating path consists of exactly one matching edge and each alternating cycle is an alternating girth. We show that finding such a solution is $\mathcal{NP}\mathcal{}$-complete, even if $g^*(G^*)=3$, by reducing independent set to it.
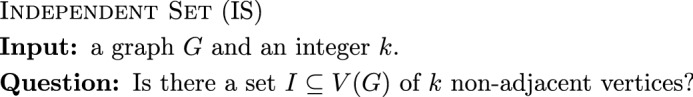


IS is $\mathcal{NP}\mathcal{}$-complete in general graphs. In order to build our reduction, we need *G* to be subcubic and triangle-free (i.e. $\Delta (G)\le 3$ and $g(G) > 3$). Note that Lozin et Milanič [12] showed that independent set remains $\mathcal{NP}\mathcal{}$-complete in $\mathcal {F}$-free planar subcubic graphs if $\mathcal {F}$ does not contain a tree with exactly three leaves. By choosing $\mathcal {F}:=\{C_3\}$ (where $C_3$ is the cycle on three vertices), we obtain the desired $\mathcal{NP}\mathcal{}$-completeness. Our reduction uses the following construction.

### Construction 1

**Fig. 3:**
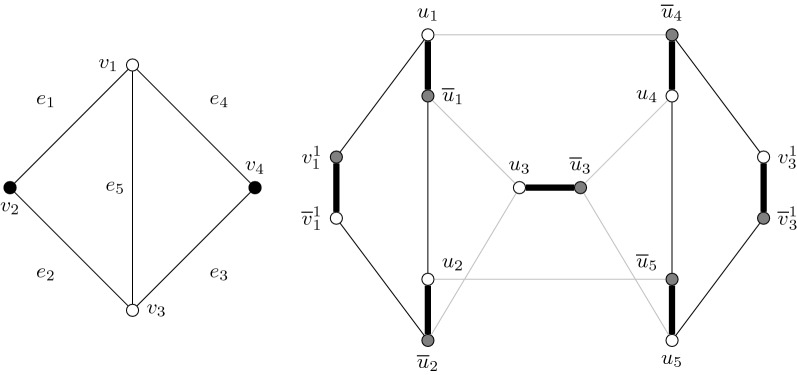
Example of a scaffold graph produced by Construction 1. Left: input graph with an independent set of size two given by the black vertices. Right: output graph with a collection of two alternating cycles and one alternating path in black. A bipartition is given by gray and white vertices. An example of a vertex-cycle is $C_{v_1}=\{\overline{v}^1_1,v^1_1,\overline{u}_1,u_1,\overline{u}_2,u_2\}$. It is possible to turn this graph into a planar graph by replacing the edges $\{u_3\overline{u}_2,u_2\overline{u_3},u_5\overline{u}_3\}$ with $\{u_3u_2,u_5\overline{u_2},\overline{u}_5\overline{u}_3\}$

(see Fig. 3) Given a subcubic, triangle-free graph *G*, construct a scaffold graph $(G^*,M^*,\omega )$ as follows:

- for each edge $e_i \in E(G)$, construct a matching edge $u_i\overline{u}_i$, and
- for each vertex $v_t \in V(G)$, introduce the matching edges $\{u^j_t\overline{u}^j_t \mid j \le 3-deg(v_t)\}=:E_t$ and construct an alternating 6-cycle $C_t$ on the vertices $E_t \cup \{u_i\overline{u}_i \mid v_t \in e_i\}$ such that no two *u* (or $\overline{u}$) vertices are adjacent.

The alternating cycles $C_i$ are called *vertex-cycles*. A bipartition is given by the *u*- and $\overline{u}$-vertices. Note that, if *G* is planar, it is also possible to construct a planar graph (which may no longer be bipartite). To show hardness of scaffolding when $|M^*|= g^*(G^*)\cdot \sigma _c + \sigma _p$, we use the following properties of graphs resulting from Construction 1.

### Lemma 1

Let *G* be a subcubic triangle-free graph and let $(G^*,M^*,\omega )$ be its scaffold graph produced by Construction 1. Let *S* be a collection of $\sigma _c = k$ alternating cycles and $\sigma _p= |M^*|-3k$ alternating paths. Then,

- $$g^*(G^*)=3$$,
- every alternating cycle in *S* is a vertex-cycle, and
- let $C_t$ and $C_{t'}$ be vertex-cycles in *S*, the vertices $v_t$ and $v_{t'}$ are not adjacent in *G*.

### Proof

- By construction, each vertex-cycle contains exactly three matching edges and, thus, $g^*(G^*) \le 3$. Suppose there is an alternating cycle containing exactly two matching edges *e* and $e'$. Let $C_t$ be a vertex-cycle containing *e*. Since $C_t$ has length six, there is another vertex-cycle $C_{t'}\ne C_t$ that contains $e'$. Indeed, *e* and $e'$ are both in $C_t$ and $C_{t'}$ since, otherwise, their extremities cannot be adjacent. By construction, there are two edges $e_i$ and $e_j$ in *G* that are incident to both $v_t$ and $v_{t'}$, contradicting *G* being simple. Hence, there is no alternating cycle with two matching edges and $g^*(G^*)=3$.
- Let *C* be an alternating cycle in *S*. By Lemma 1(a), $|M^*| = g^*(G^*)\cdot \sigma _c + \sigma _p$, implying that *C* has length six. Let $u_i\overline{u}_i$ be a matching edge of *C*. If there is a matching edge $v^1_t\overline{v}^1_t \in C$ then, by construction, the third matching edge of *C* is either $v_t^2\overline{v}_t^2$ (if $deg(v_t)=1$) or $u_j\overline{u}_j$ (where $v_t \in e_j$ in *G*). Thus, *C* is the vertex-cycle $C_t$. Suppose there is no matching edge $v^1_t\overline{v}^1_t$ in *C*. For any pair of matching edges $(u_k\overline{u}_k,u_{k'}\overline{u}_{k'})$ of *C*, $e_k$ and $e_{k'}$ are incident to a same vertex in *G*. Let $u_i\overline{u_i},u_j\overline{u_j}$ and $u_k\overline{u_k}$ be the three matching edges of *C*. Since *G* is triangle-free, $e_i, e_j$ and $e_k$ are adjacent in *G*, hence, *C* is a vertex-cycle.
- Let $e_i = v_tv_{t'}\in E(G)$. The matching edge $u_i\overline{u}_i$ is in $C_t$ and $C_{t'}$ and, thus, *S* cannot contain both $C_t$ and $C_{t'}$.

□

In the proof of correctness, we simulate vertices of the independent set with vertex-cycles. If a solution *S* contains two vertex cycles $C_i$ and $C_j$, then $v_i$ and $v_j$ are not adjacent in *G*. Hence, if a solution *S* contains *k* vertex-cycles, then there is an independent set of *k* vertices in *G*.

### Theorem 2

scaffolding is $\mathcal{NP}\mathcal{}$-complete, even in bipartite (or planar) subcubic scaffold graphs $(G^*,M^*,\omega )$ were $|M^*| = g^*(G^*) \cdot \sigma _c + \sigma _p$ and $g^*=3$.

### Proof

Since, clearly, scaffolding is in $\mathcal{NP}\mathcal{}$, it remains to show that Construction 1 is a reduction, that is, *G* has an independent set of size *k* if and only if there is a collection of *k* alternating cycles and $|M^*|-3k$ alternating paths in $(G^*,M^*)$.

“$\Rightarrow$”: Let *I* be an independent set of size *k* in *G*. We build a solution of scaffolding as follows. For each vertex $v_t \in I$, we construct the vertex-cycle $C_t$ in *S*. For each remaining matching edge in $M^*\setminus \bigcup _{v_t\in I}C_t$, we construct an alternating path of length one. We obtain a solution *S* as thought.

“$\Leftarrow$”: Let *S* be a solution in $(G^*,M^*)$ containing *k* alternating cycles and $|E(G)-k$ alternating paths and let $I := \{v_t | C_t \in S\}$. By Lemma 1(b), any alternating cycle of *S* is a vertex-cycle in $(G^*,M^*)$ and, thus, $|I|=k$. Moreover, by Lemma 1(c), *I* is independent in *G*.□

Note that Theorem 2 can be generalized to $g^*(G^*)>3$ by modifying Construction 1 as follows. First, we build our construction from a graph *G* with $g(G)>\ell \ge 3$. IS remains $\mathcal{NP}\mathcal{}$-complete in such graphs by the result of Lozin and Milanič: it suffices to take $F=\{C_{i} \mid i \le \ell \}$, where $C_i$ is the cycle of order *i*. Then, we increase the length of every vertex-cycle by taking $E_t = \{u^j_t\overline{u}^j_t \mid j\le 3+\ell - deg(v_t)\}$ for each $v_t \in V(G)$. By making these modifications, we construct a scaffold graph with $g^*(G^*) = \ell$ and we preserve properties Lemma 1(b) and Lemma 1(c). This leads to the following result.

### Corollary 1

scaffolding is $\mathcal{NP}\mathcal{}$-complete even in bipartite (or planar) subcubic scaffold graphs $(G^*,M^*)$ were $|M^*| = g^*(G^*) \cdot \sigma _c + \sigma _p$, for all $g^*(G^*)\ge 3$.

## Non-approximability

In this section, we discuss the hardness of approximating max scaffolding. Notice that, since scaffolding is $\mathcal{NP}\mathcal{}$-complete, there is no polynomial-time approximation algorithm for max scaffolding (unless $\mathcal {P} =\mathcal{NP}\mathcal{}$). However, this argument does not hold for graph classes where scaffolding is in $\mathcal {P}$ (*i.e.* classes for which the feasibility function (and, thus, the greedy algorithm) runs in polynomial time).

We show that, in this case, max scaffolding is still Poly-APX-hard, that is, it is not possible to approximate max scaffolding within a factor better than a polynomial function in $|V(G^*)|+|E(G^*)|$ (unless $\mathcal {P} =\mathcal{NP}\mathcal{}$). Recall that Fig. 2 already shows that the greedy algorithm can not approximate max scaffolding with a ratio better than a polynomial function. The inapproximability result presented in this section shows that it is the case for any polynomial-time algorithm. In the following, we construct an *S*-reduction (see [13]) from the optimization version of independent set.

### Construction 2

**Fig. 4:**
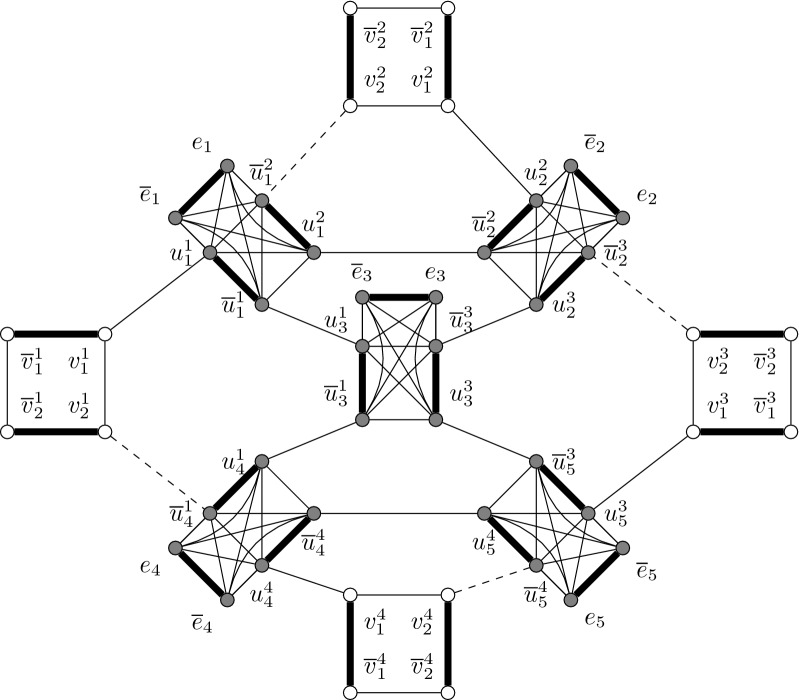
Example of a scaffold graph produced by Construction 2. The input graph is composed by the edges $e_1 = v_1v_2$, $e_2 = v_2v_3$, $e_3= v_1v_3$, $e_4= v_1v_4$ and $e_5 = v_3v_4$. Gray vertices in the figure belong to an edge gadget and white vertices belong to a vertex gadget. Matching edges are bold. Solid edges have weight zero and dashed edges have weight one. The long vertex-cycle $C(v_2)$ corresponds to the vertices $\{\overline{v}^2_1,v^2_1,u^2_2,\overline{u}^2_2,u^2_1,\overline{u}^2_1,v^2_2,\overline{v}^2_2\}$

(see Fig. 4) Let *G* be a graph. Then, construct the following scaffold graph $(G^*,M^*,\omega )$:

- For each $e_i= v_tv_q\in E(G)$, construct a clique $\{u_i^t,\overline{u}_i^t,u^q_i,\overline{u}_i^q,e_i,\overline{e}_i\}$ with $u_i^t\overline{u}_i^t,u_i^q\overline{u}_i^q,e_i\overline{e}_i \in M^*$.
- For each $v_t\in V(G)$, construct a cycle $(v^t_1,\overline{v}^t_1,\overline{v}^t_2,v^t_2)$ with $v^t_1\overline{v}^t_1,v^t_2\overline{v}^t_2 \in M^*$.
- Let $v_t \in V(G)$ and let $\mathcal {A}_t$ be a list of all edges incident with $v_t$ in *G*. Construct an alternating cycle containing all vertices in $\{v^t_1,\overline{v}^t_1,v^t_2,\overline{v}^t_2\} \cup \{ u^t_i,\overline{u}^t_i \mid \forall e_i \in \mathcal {A}_t\}$ as follows:
  - For all $k < d(v_t)$, let $e_i$ and $e_j$ be the $k^\text {th}$ and $k+1^\text {st}$ edges of $\mathcal {A}_t$, respectively, and add a non-matching edge between $\overline{u}^t_i$ and $u^t_j$.
  - Let $e_i$ and $e_j$ be the first and last edges of $\mathcal {A}_t$, respectively, and add the non-matching edges $v^t_1u^t_i$ and $v^t_2\overline{u}^t_j$.
- Each non-matching edge has weight zero, except the edges $v^t_2\overline{e}_j$ which have weight one.

Let $v_t\in V(G)$. The cycle on $\{v^t_1,\overline{v}^t_1,v^t_2,\overline{v}^t_2\} \cup \{u^t_i,\overline{u}^t_i \mid \exists q\; e_i = v_tv_q \in E(G)\}$ is called the *long vertex-cycle* of $v_t$ and is denoted by $C(v_t)$. Note that a long vertex-cycle has weight one. Now consider the following properties.

### Lemma 2

Let *G* be a graph and let $(G^*,M^*,\omega )$ be the scaffold graph produced by Construction 2. Let *S* be a collection of $|V(G)|+|E(G)|$ alternating cycles in $(G^*,M^*,\omega )$.

- Every non-zero-weight alternating cycle *C* of *S* is a long vertex-cycle.
- Let $C(v_t)$ and $C(v_q)$ be two long vertex-cycles of *S*. Then, $v_tv_q\notin E(G)$.

### Proof

Note that it is always possible to build a collection of $|V(G)|+|E(G)|$ (weight-0) alternating cycles in $(G^*,M^*,\omega )$ by constructing the alternating cycle $\{u^t_i, \overline{u}^t_i,u^q_i,\overline{u}^q_i,e_i,\overline{e}_i\}$ for each edge $e_i=v_tv_q$ of *G* and the alternating cycle $\{v^t_1,\overline{v}^t_{1},v^t_2,\overline{v}^t_2\}$ for each vertex $v_t\in V(G)$.□

### Claim 1

Let $v_t\in V(G)$ and $e_i\in E(G)$. Then, no alternating cycle of *S* contains both $e_i\overline{e}_i$ and $v^t_1\overline{v}^t_1$.

### Proof

Towards a contradiction, assume that there is such an alternating cycle *C*. By pidgeonhole principle, one of the $|V(G)+E(G)|$ alternating cycles in *S*, say $C'$, avoids both $e_i\overline{e}_i$ and $v^t_1\overline{v}^t_1$ for all $i,t\in \mathbb {N}$. Let $u^t_i\overline{u}^t_i$ be a matching edge of $C'$ for some $e_i=v_tv_q$. Then, $C'$ cannot contain $u^q_i\overline{u}^q_i$ as, otherwise, $e_i\overline{e}_i$ cannot be part of an alteranting cycle in *S*, implying that *S* is not a solution. Thus, each matching edge of $C'$ is on the long vertex-cycle $C(v_t)$. Since the graph induced by the vertices of $C(v_t) \setminus v^t_1\overline{v}^t_1$ is a path, it is not possible to construct $C'$. Hence, we conclude that *C* does not exist.□

(a): Let *C* be a non-zero-weight alternating cycle of *S* and assume towards a contradiction that *C* is not a long vertex-cycle. Since *C* contains a non-zero-weight edge $v^t_2\overline{u}^1_i$, the matching edge $v^t_2\overline{v}^t_2$ is in *C*. As *C* is not a long vertex-cycle, there is some $e_i=v_tv_q$ such that *C* contains both $u^t_i\overline{u}^t_i$ and $u^q_i\overline{u}^q_i$. Thus, either the matching edge $e_i\overline{e}_i$ is in *C*, contradicting Claim 1, or $e_i\overline{e}_i$ consists of a single-edge alternating path of *S*, contradicting our choice of *S*.

(b): Towards a contradiction, assume that *S* contains $C(v_t)$ and $C(v_q)$ such that $e_i=v_tv_q\in E(G)$. Then, the matching edge $e_i\overline{e}_i$ is a single-edge alternating path of *S*, contradicting our choice of *S*.

We now show the Poly-APX-hardness of max scaffolding, even for graph classes for which $\textsc {Scaffolding} \in \mathcal {P}$. Reusing the same idea of Theorem 2, we simulate the vertices of the independent set with long vertex-cycles. If a solution *S* of max scaffolding has weight *k*, then *S* contains *k* long vertex-cycles and, since their related vertices cannot be adjacent, we can construct an independent set with *k* vertices in *G*.

### Theorem 3

max scaffolding is Poly-APX-hard, even for graph classes for which $\textsc {Scaffolding} \in \mathcal {P}$.

### Proof

Let *G* be an instance of independent set and let $(G^*,M^*,\omega )$ be the scaffold graph produced by Construction 2. Let $\mathcal {S}$ be the set of all collections of $\sigma _p=0$ alternating paths and $\sigma _c = |V(G)|+|E(G)|$ alternating cycles in $(G^*,M^*,\omega )$.

Recall that independent set is Poly-APX-complete for general graphs [14]. We show that *G* has a size-*k* independent set if and only if $\mathcal {S}$ contains a solution *S* of score *k*.

“$\Rightarrow$”: Let *I* be an independent set of size *k* in *G*. We construct a solution $S\in \mathcal {S}$ as follows.

First, for each $v_t\in I$, construct the alternating cycle $C(v_t)$ in *S*. Second, for each $v_t\in V(G)\setminus I$, construct the alternating cycle $(v^t_1,\overline{v}^t_{1},\overline{v}^t_2,v^t_2)$ in *S*.

Third, for each edge $e_i=v_tv_q$ not incident with a vertex in *I*, construct the alternating cycle $(u^t_i, \overline{u}^t_i,\overline{u}^q_i,u^q_i,e_i,\overline{e}_i)$ in *S*.

Fourth, for each edge $e_i=v_tv_q$ with $v_t\in I$, (the matching edge $u^t_i\overline{u}^t_i$ is in $C(v_t)$ which is already in *S*), construct the alternating cycle $(u^q_i,\overline{u}^{q}_i,e_i,\overline{e}_i)$.

Since each long vertex-cycle has weight one, we obtain a solution *S* with $\omega (S)= k$.

“$\Leftarrow$”: Let $S\in \mathcal {S}$ with $\omega (S) = k$. We construct an independent set *I* by taking all vertices whose long vertex-cycle is in *S*, that is, $I := \{ v_t \mid C(v_t) \in S\}$. Since each long vertex-cycle has weight one, Lemma 2a implies that *S* contains *k* long vertex-cycles. Thus, $|I| = k$. Further, by Lemma 2b, *I* is independent.

Let *f* be the function corresponding to Construction 2 and let *g* be a function that computes an independent set in *G* from a solution in *f*(*G*), as described above. Suppose that there is a polynomial-time algorithm *A* with approximation factor $\rho$ for max scaffolding. The approximation factor of $g \circ A \circ f$ is equal to $\rho$, thus Construction 2 constitutes an *S*-reduction. Non-approximability results of independent set transfer to max scaffolding.□

## Feasibility function for connected cluster graphs

In this section, we present a feasibility function for connected cluster graphs using dynamic programming. For simplicity, we consider in the following scaffold graphs $(G^*,M^*,\omega )$ such that $G^*$ is a connected cluster graph and no matching edge is a bridge. The case were a bridge can be a matching edge is included in the feasibility function for block graph that (see "Experimental results" section).

### Definitions

Notice that the structure of a connected cluster graph is close to a tree (that is, collapsing each clique of $G^*$ into a single vertex leads to a tree), so we will use a similar vocabulary: a *rooted* connected cluster graph is a connected cluster graph where a clique *r* is designated as a *root*. Then, the following notation applies: the *parent* of a clique *x* is the clique connected to *x* on the unique *x*-*r*-path. A *child* of a clique *c* is clique of which *c* is the parent. Any clique without children is called a *leaf*. A vertex *v* of a clique *c* is a *door* of *c* if *v* is adjacent to a vertex *u* in a child of *c*. In that case, for simplicity, we say that the clique containing *u* is a child of *v*. The *upper door* of a clique $c\ne r$ is the unique vertex *v* that is adjacent to a vertex of the parent of *c*. Let *c* be a clique of $G^*$ and let *S* be a partial solution in $G^*$. Let $S'$ be the intersection of *S* and *c*, an *alternating element* of *c* is either an alternating cycle of $S'$ or an alternating path of $S'$. Notice that an alternating path of *S* can be decomposed into several alternating elements if it belongs to several cliques. Let *e* be the alternating element containing the upper door of *c*. The *subclique* $c'$ of *c* is the subgraph containing every vertex of *c* that does not belong to *e*. Formally, $c'=G^*[V(c)\setminus V(e)]$. We use the tree-structure to develop a bottom-up algorithm, that is, we construct and assemble some partial solutions from the leaves to the root. We define some operations to combine this partial solutions.

### Operations

Let $G_1$ and $G_2$ be two edge-disjoint subgraphs. We can build a solution in the graph induced by $V(G_1) \cup V(G_2)$ from a solution in $G_1$ and a solution in $G_2$, using four operations.

#### Definition 2

Let $G_1$ and $G_2$ be edge-disjoint subgraphs of $G^*$. Let $S_1$ and $S_2$ be solutions of $G_1$ and $G_2$, respectively. Let *S* be a solution of $G^*[V(G_1) \cup V(G_2)]$. *S* is a *composition* of $S_1$ and $S_2$ if *S* can be obtained from $S_1\cup S_2$ by at most one of the following operations:

Merger: merge an alternating path $(u_1,u_2,\ldots ,u_{2t})$ of $S_1$ with an alternating path $(v_1,v_2,\ldots ,v_{2q})$ of $S_2$ by adding the non-matching edge $u_{2t}v_1$.
Closing: close an alternating path $(u_1,u_2,\ldots ,u_{2t})$ of $S_1$ and an alternating path $(v_1,v_2,\ldots , v_{2q})$ of $S_2$ into an alternating cycle by adding the non-matching edges $u_{2t}v_1$ and $v_{2q}u_1$.
Absorption: replace a non-matching edge $v_{2i}v_{2i+1}$ of an alteranting path in $S_2$ with an alternating path $(u_1,u_2,\ldots ,u_{2t}$ of $S_1$ by removing $v_{2i}v_{2i+1}$ and adding the non-matching edges $v_{2i}u_1$ and $u_{2t}v_{2i+1}$. We call $v_{2i}v_{2i+1}$ *absorbent*.

Finally, if no operation is necessary to obtain *S* from $S_1\cup S_2$, we say that *S* is obtained by **juxtaposition**.

Note that all presented operations add only edges of $E(G^*) \setminus (E(G_1) \cup E(G_2))$. Note further that not all compositions of two solutions are guaranteed to exist for a pair $S_1$ and $S_2$. In the algorithm, we manipulate sets of solutions: we can create a new set of solutions from two sets of solution if all pairs of solutions of the two input sets are used in the resulting set.

#### Definition 3

Let $G_1$ and $G_2$ be two edge-disjoint subgraphs of $G^*$ and let $\mathcal {S}_1$ and $\mathcal {S}_2$ be sets of solutions of subgraphs $G_1$ and $G_2$, respectively. Let *op* be one the operation described in Definition 2. Then, we call the set $\mathcal {S}=\{op(S_1,S_2)\mid \forall S_1\in \mathcal {S}_1 \wedge \forall S_2\in \mathcal {S}_2\;\}$ the *complete composition* of $\mathcal {S}_1$ and $\mathcal {S}_2$.

To ensure the possibility of building a complete composition from two sets of solutions, it is useful to characterize a solution according to the operations we can perform on it.

#### Definition 4

Let *G* and $G'$ be two edge-disjoint subgraphs of $G^*$ and let *S* be a feasible solution of scaffolding for $(G,M^*,\omega )$.

1. *S* is *closeable* if *S* contains an alternating path $(u_1,u_2\ldots ,u_{2t})$ and $G'$ contains an alternating path $(v_1,v_2,\ldots ,v_{2q})$ such that $u_{2t}v_1$ and $v_{2q}u_1$ are edges of $E(G^*\setminus M^*$.
2. *S* is *extensible* by $G'$ if *S* contains a vertex *v* such that *v* is an extremity of an alternating path and *v* has a neighbor in $G'$ .
3. *S* is *frozen* to $G'$ if *S* is not extensible.
4. *S* is *absorbent* to $G'$ if *S* contains an alternating path $(u_1,u_2,\ldots ,u_{2t})$ and $G'$ contains an alternating path with extremities *v* and *w* such that $vu_{2i}, wu_{2i+1} \in E(G^*)\setminus M^*$ for some $i<t$. Note that an absorbent solution can also be closeable, alternating or frozen.

When omitted, $G'$ defaults to $G^*- V(G)$.

**Fig. 5:**
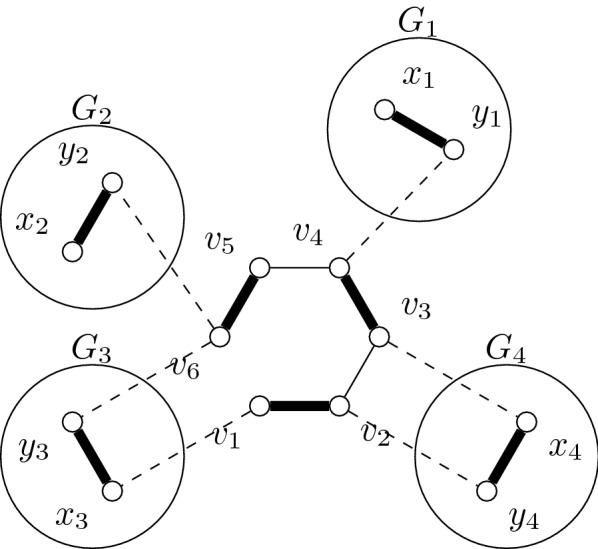
The solution *S* is composed of a single alternating path $\{v_1,\dots ,v_6\}$. *S* is closeable by subgraph $G_3= \{x_3,y_3\}$: we can close the alternating path of *S* into an alternating cycle by adding the edges $v_1x_3$, $x_3y_3$ and $y_3v_6$. *S* is extensible by subgraph $G_2 = \{x_2,y_2\}$: we can extend the alternating path of *S* by adding the edges $v_6y_2$ and $y_2x_2$ without changing the number of paths in *S*. *S* is absorbent to $G_4 = \{x_4,y_4\}$: we can replace the edge $v_2v_3$ of *S* by the edges $v_2y_4,y_4x_4$ and $x_4v_3$ without changing the number of paths in *S*. *S* is frozen to $G_1= \{x_1,y_1\}$

Note that all closeable solutions are also extensible. If a solution *S* is closeable by a subgraph $G'$, then we can close an alternating path of *S* into an alternating cycle by adding some edges of $G'$. If a solution *S* is extensible by a subgraph $G'$, then we can add some edges of $G'$ in an extremity of an alternating path of *S* without changing the cardinality of the solution. Finally, if a solution *S* is absorbent to a subgraph $G'$, then we can replace an absorbent edge of *S* by a path of length three without changing the cardinality of *S*. An example of the different operations of Definition 4 is given in Fig. 5.

### Semantics

Since the number of possible solutions can be exponential, we just store the possible cardinalities in the table entries, which is sufficient to answer the question of feasibility. Recall that, if $X, Y \subseteq \mathbb {N}$ are two sets of integers, then the sum of *X* and *Y* is defined as $X+Y = \{ x+y\mid x \in X, y \in Y \}$. Note that $X + \varnothing = \varnothing$. In the following, we call an integer *j* *eligible* with respect to a set $\mathcal {S}$ of solutions and an integer *i* if there is a solution $S \in \mathcal {S}$ containing *i* alternating cycles and *j* alternating paths. Then, our dynamic programming table has the following semantics.

#### Definition 5

(Semantics) Let $\mathcal {S}$ be a set of solutions and let $i \in \mathbb {N}$.

A table entry $[\mathcal {S},i]$ is the set of all integers eligible with respect to $\mathcal {S}$ and *i*. More formally, letting $X_i = \{S \mid S \in \mathcal {S} \wedge \sigma _c(S) = i\}$, we define $[\mathcal {S},i] = \{\sigma _p(S) \mid S \in X_i\}$.

Let us highlight three particular values of $[\mathcal {S},i]$. For $\mathcal {S}=\{\varnothing \}$, we have $[\{\varnothing \},0] = \{0\}$ and, for each $i>0$, we have $[\{\varnothing \},i]=\varnothing$. For an alternating path *p*, we have $[\{p\},0] = \{1\}$ and $[\{p\},i]=\varnothing$ for each $i>0$. Finally, for an alternating cycle *c*, we have $[\{c\},1] = \{0\}$ and $[\{c\},i]=\varnothing$ for each $i\ne 1$. For brevity, we let $[\mathcal {S}]$ denote the vector $([\mathcal {S},0],\dots ,[\mathcal {S},\sigma _c])$ and, for any operator $\diamond$ and any sets $\mathcal {S}_1$ and $\mathcal {S}_2$ of solutions, we define $[\mathcal {S}_1] \diamond [\mathcal {S}_2]$ as component-wise $\diamond$, that is, $[\mathcal {S}_1,i] \diamond [\mathcal {S}_2,i]$ for each $i \in [0,\sigma _c]$.

#### Lemma 3

Let $G_1$ and $G_2$ be two vertex-disjoint subgraphs of $G^*$ and let $\mathcal {S}_1$ and $\mathcal {S}_2$ be sets of solutions of $G_1$ and $G_2$, respectively. Let $\mathcal {S}$ be a set of solutions of $G^*[V(G_1) \cup V(G_2)]$ such that $\mathcal {S}$ is a complete composition of $\mathcal {S}_1$ and $\mathcal {S}_2$.

1. If $\mathcal {S}$ is the set of solutions composed with a merger operation, then $[\mathcal {S},k] = \bigcup _{i+j=k}([\mathcal {S}_1,i] + [\mathcal {S}_2,j] + \{-1\})$.
2. If $\mathcal {S}$ is the set of solutions composed with a closing operation, then $[\mathcal {S},k] = \bigcup _{i+j+1=k}([\mathcal {S}_1,i] + [\mathcal {S}_2,j] + \{-2\})$.
3. If $\mathcal {S}$ is the set of solutions composed with an absorption operation, then $[\mathcal {S},k] = \bigcup _{i+j=k}([\mathcal {S}_1,i] + [\mathcal {S}_2,j] + \{-1\})$.
4. If $\mathcal {S}$ is the set of solutions composed with a juxtaposition operation, then $[\mathcal {S},k] = \bigcup _{i+j=k}([\mathcal {S}_1,i] + [\mathcal {S}_2,j])$.

#### Proof

Let $S \in \mathcal {S}$ and let $S_1$ and $S_2$ denote the solutions of $\mathcal {S}_1$ and $\mathcal {S}_2$, respectively, such that *S* is composed by $S_1$ and $S_2$. Then,

1. since $S_1$ and $S_2$ have a common alternating path in *S*, we have $\sigma _p(S) = \sigma _p(S_1) + \sigma _p(S_2) -1$ and since no cycle is formed, $\sigma _c(S) = \sigma _c(S_1) + \sigma _c(S_2)$. Thus, since $\mathcal {S}$ is a complete composition of $\mathcal {S}_1$ and $\mathcal {S}_2$, we have $[\mathcal {S},k] = \bigcup _{i+j=k}([\mathcal {S}_1,i] + [\mathcal {S}_2,j] + \{-1\})$.
2. since one path of $S_1$ and one path of $S_2$ are closed into a single alternating cycle, we have $\sigma _p(S) = \sigma _p(S_1) + \sigma _p(S_2) -2$ and $\sigma _c(S) = \sigma _c(S_1) + \sigma _c(S_2) + 1$. Thus, since $\mathcal {S}$ is a complete composition of $\mathcal {S}_1$ and $\mathcal {S_2}$, we have $[\mathcal {S},k] = \bigcup _{i+j=k}([\mathcal {S}_1,i] + [\mathcal {S}_2,j] + \{-2\})$.
3. since $S_1$ has an alternating path that is “absorbed” into a connected component of $S_2$, we have $\sigma _p(S) = \sigma _p(S_1) + \sigma _p(S_2) -1$ and since no cycle is formed, $\sigma _c(S) = \sigma _c(S_1) + \sigma _c(S_2)$. Thus, since $\mathcal {S}$ is a complete composition of $\mathcal {S}_1$ and $\mathcal {S_2}$, we have $[\mathcal {S},k] = \bigcup _{i+j=k}([\mathcal {S}_1,i] + [\mathcal {S}_2,j] + \{-1\})$.
4. since all paths and cycles of $S_1$ and $S_2$ are present in *S*, we have $\sigma _p(S) = \sigma _p(S_1) + \sigma _p(S_2) -1$ and since no cycle is formed, $\sigma _c(S) = \sigma _c(S_1) + \sigma _c(S_2)$. Thus, since $\mathcal {S}$ is a complete composition of $\mathcal {S}_1$ and $\mathcal {S_2}$, we have $[\mathcal {S},k] = \bigcup _{i+j=k}([\mathcal {S}_1,i] + [\mathcal {S}_2,j])$.

$$\square$$

We use Lemma 3 to define the four functions *juxtapose*, $merge_t$, *absorb*, and $close_t$, which provide table entries for complete compositions “composed” with a juxtaposition, merge, absorption or closing operation, respectively. Although Lemma 3 is defined for two sets, we use a generalized version which can take as parameters more than two sets. The functions $merge_t$ and $close_t$ have a parameter *t* that indicates the number of paths merged or closed during the operation. For example, if we have three sets $S_1$, $S_2$, and $S_3$ and if it is possible to construct a single alternating path in the resulting composition by taking one alternating path in each set, then we use the function $merge_3(\{S_1\},\{S_2\},\{S_3\})$. Note that the parameter *t* can be different from the number of sets. In addition, it is sometimes possible to close a single alternating path into an alternating cycle and, in that case, the function $close_1$ is used. The four functions are defined in Algorithm 2, Algorithm 3 and Algorithm 4. However, we must ensure that the associated operation is feasible before using one these functions.
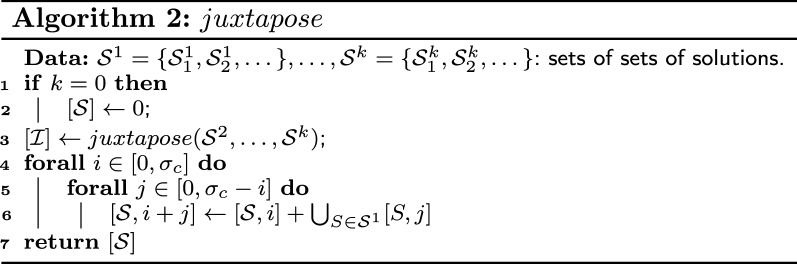

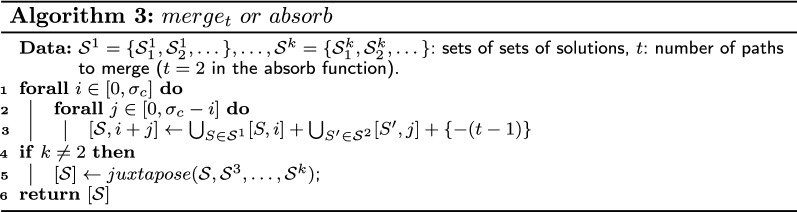

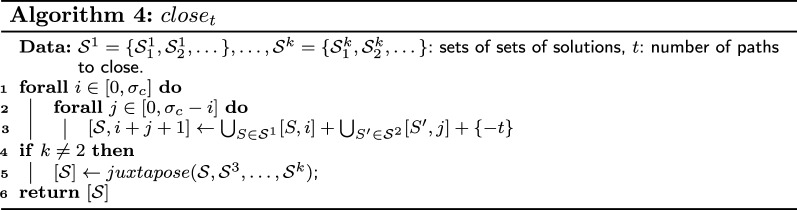


In the algorithm, we traverse four different types of subgraphs defined as follows.

- Let $v \in V(G^*)$, let *child*(*v*) be the set of children of *v* in $G^*$ (possibly empty). Then, $G^*(v)$ denotes the subgrah of $G^*$ that is induced by *v* and every branch linked to *v*. Formally, $G^*(v) := G^*[\{v\} \cup \bigcup \limits _{x \in child(v)} V(G^*(x))]$.
- Let *e* be an alternating element. Then, $G^*(e)$ denotes the subgraph of $G^*$ that is induced by *e* and all children of its vertices. Formally, $G^*(e) = G^*[\bigcup \nolimits _{v \in e} V(G^*(v))]$.
- Let *c* be a clique of $G^*$ and let $c'$ be the subclique of *c*. For all $x \in \{c,c'\}$, the subgraph $G^*(x)$ is the union of *x* and all children of *x*. Formally, $$G^*(x)=G^*[\bigcup \nolimits _{e \in M^*\cap ( {\begin{array}{c}x\\ 2\end{array}}) } V(G^*(e))]$$.

For each traversed subgraph, we use four different sets of solutions distinguishing solutions according to their properties.

#### Definition 6

Let *S* be a partial solution of $G^*$. Let *x* be a vertex, a partial path, a subclique or clique of $G^*$ and let $S'$ be a solution of the subgraph $G^*(x)$. Then,

- $S \in \mathcal {C}(x) \Leftrightarrow$ $S'$ is closeable and $S \cap E(G^*(x)) \subseteq S'$.
- $S \in \mathcal {E}(x) \Leftrightarrow$ $S \notin \mathcal {C}(x)$ and *S* is extensible and $S \cap E(G^*(x)) \subseteq S'$.
- $S \in \mathcal {A}(x) \Leftrightarrow$ *S* is frozen and absorbent and $S \cap E(G^*(x)) \subseteq S'$.
- $S \in \mathcal {F}(x) \Leftrightarrow$ $S \notin \mathcal {A}(x)$ and *S* is frozen and $S \cap E(G^*(x)) \subseteq S'$.

### The algorithm

We now present a method to provide the feasibility function needed by Algorithm 1. In the next paragraphs, we describe the algorithms that calculate the table entries for the four types of subgraphs described above.

#### Vertex

**Fig. 6:**
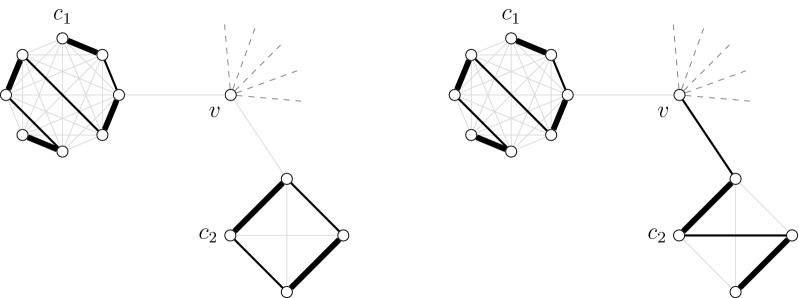
Example of two solutions $S_1$ (left, frozen) and $S_2$ (right, extensible) in $G^*(v)$. The cliques $c_1$ and $c_2$ are children of *c*(*v*). Each of $S_1$ and $S_2$ contains two alternating elements (solid black lines). The frozen solution is obtained with the juxtaposition of two frozen solutions in $c_1$ and $c_2$. The extensible solution is obtained with the juxtaposition of a frozen solution in $c_1$ and with the merge between *v* and an extensible solution in $c_2$

Let $v\in V(G^*)$. We show in this part how to compute the table entries for the sets $\mathcal {F}(v)$ and $\mathcal {E}(v)$. Note that, since the edge between $G^*(v)$ and its parent is a bridge, any solution $S'$ for $G^*(v)$ can have at most one edge incident to *v*. Thus, the sets $\mathcal {C}(v)$ and $\mathcal {A}(v)$ are empty. If *v* is not incident to an edge of $S \cap E(G^*(v))$, then we construct the table entries by successively merging the table entries of the children adjacent to *v*. For that, we use at each step an intermediate graph $G_i$. Let $V_i$ be the set of the first *i* children of *v*. $G_i$ is the subgraph of $G^*$ induced by *v* and all vertices in $V_i$. If *v* is incident with an edge $S \cap E(G^*(v))$, then any solution containing *S* is in $\mathcal {E}(v)$. An example of solutions computed by Algorithm 5 is depicted in Fig. 6.

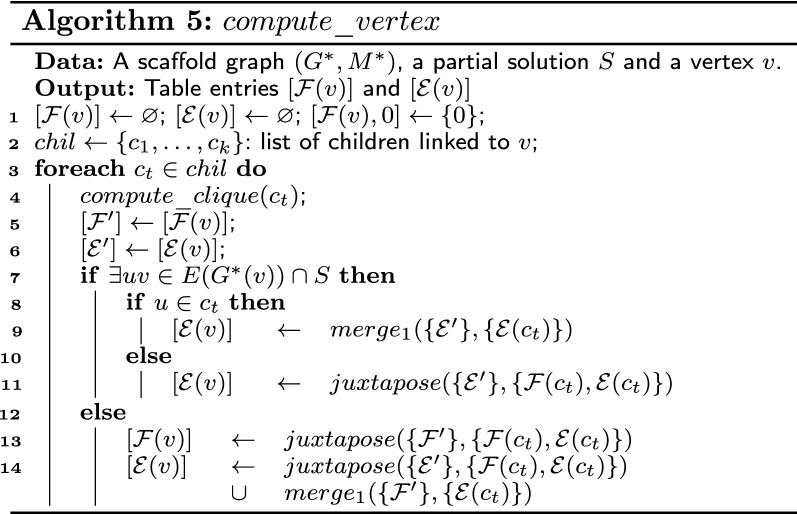



##### Lemma 4

For any vertex *v*, the values of the table entries provided by Algorithm 5 are correct for the set $\mathcal {F}(v)$ and $\mathcal {E}(v)$.

##### *Proof*

First, if there is no child linked to *v*, then $G^*(v)$ consists of the single vertex *v*. In that case, the only solution for $G^*(v)$ consists of zero alternating cycles and paths and this solution is frozen. Thus, the initial values given to $[\mathcal {F}(v)]$ and $[\mathcal {E}(v)]$ in the initialization step (i.e. lines 1 to 2) are correct. Assume that table entries returned by $compute\_clique$ are correct. Let $S'$ be a solution of $G^*(v)$ such that $S\cap E(G^*(v)) \subseteq S'$. We distinguish two cases.

**Case 1:** there is an edge $uv \in S\cap E(G^*(v))$. Thus, $S'$ is extensible and is composed by the merge of an extensible solution in $G^*(c_u)$ with *uv* and the juxtaposition of any solution for each child $c_{u'}\ne c_u$. Hence, lines 9 and 11 are correct.
**Case 2:** there is no edge $uv \in S\cap E(G^*(v))$. Then, $S'$ is frozen if and only if it does not contain an edge incident to *v*. As there is no edge *uv* in any child $c_t$, $S'$ is composed by juxtaposition of any solution for each child $c_t$ and the assignment in line 13 is correct. If $S'$ is extensible, then there is a unique child $c_t$ of *v* such that an alternating path from $S' \cap E(G^*(c_t))$ has been expanded to *v* and, therefore, the solution $S' \cap E(G^*(c_t))$ is extensible. Thus, $S'$ is composed by a merge of a extensible solution of a unique child and the juxtaposition of any solution in other children. Hence, line 14 is correct.

□

#### Alternating element

**Fig. 7:**
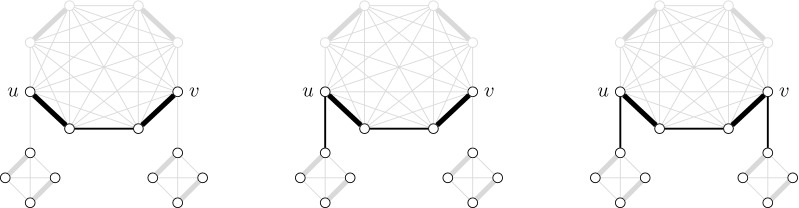
Example of solutions (black edges) in $G^*(e)$ where *e* is a *u*-*v*-path. The left solution is closeable, the center solution is extensible and the right solution is frozen. The closeable solution is obtained by the juxtaposition of *e*, any solution in $G^*(u)$ and any solution in $G^*(v)$. The extensible solution is obtained by the merge of *e* with an extensible solution in $G^*(u)$ and the juxtaposition of any solution in $G^*(v)$. The frozen solution is obtained by the merge of *e*, an extensible solution of $G^*(u)$ and an extensible solution in $G^*(v)$

Let *c* be a clique of $G^*$ and let *e* be an alternating element of *c* such that *e* does not contain the upper door of *c*. We show in this part how to compute the table entries for the sets $\mathcal {C}(e)$, $\mathcal {F}(e)$ and $\mathcal {E}(e)$. If *e* is a *u*-*v*-path, then the idea is to merge the computed table entries of *u* and *v* and juxtapose the frozen solutions of the inner vertices. If *e* is an alternating cycle, then there is no choice and the only solution containing *S* is frozen. An example of solutions computed by Algorithm 6 is depicted in Fig. 7.

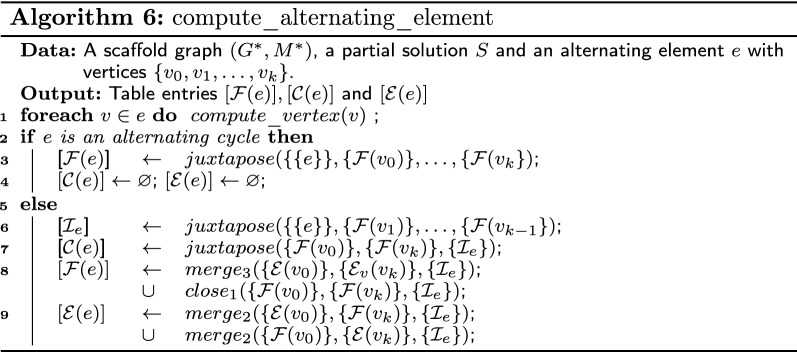



##### Lemma 5

For any alternating element *e*, the values of the table entries provided by Algorithm 6 are correct for the sets $\mathcal {C}(e)$, $\mathcal {F}(e)$ and $\mathcal {E}(e)$.

Note that the only possibility to obtain an absorbent solution of $G^*(e)$ is when *e* is a path that is closed into an alternating cycle. However, if an absorption operation is done in the function $compute\_subclique$, then the resulting solution can also be obtained by a closing operation with a solution in $\mathcal {C}(e)$. Thus, our dynamic programming will not compute the value of $[\mathcal {A}(e)]$.

##### Proof

Suppose that the values of the table entries provided by the function $compute\_vertex$ are correct. First note that, for each inner vertex $v_t$ of *e*, the subsolutions of $G^*(v_t)$ are necessarily frozen, then a solution of $G^*(e)$ contains a juxtaposition of frozen solutions of the inner vertices of *e*. If *e* is an alternating cycle, then the only possible solution is obtained by the juxtaposition of frozen solutions of the inner vertices and the alternating cycle *e*. Thus, the assignment line 5 is correct. Suppose that *e* is a partial path. All possible values of the juxtaposition of the frozen solutions of the inner vertices are assigned in the table entry $[\mathcal {I}_e]$.

- A solution $S'$ of $G^*(e)$ is closeable if the degree of the extremities of *e* are equal to one. Then, the subsolutions of $S'$ in $G^*(v_0)$ and $G^*(v_k)$ are frozen. Thus, the assignment line 7 is correct.
- A solution $S'$ of $G^*(e)$ is frozen if the degree of the extremities of *e* are equal to two. It is the case if (1) the subsolutions of $S'$ in $G^*(v_0)$ and $G^*(v_k)$ are extensible or (2) the subsolutions of $S'$ in $G^*(v_0)$ and $G^*(v_k)$ are frozen and *e* is closed into an alternating cycle. Thus, the assignment line 8 is correct.
- A solution $S'$ of $G^*(e)$ is extensible and not closeable if and only if exactly one vertex in $\{v_0,v_k\}$ has degree one. Then, exactly one subsolution of *S* in $G^*(v_0)$ or $G^*(v_k)$ is extensible. Thus, the assignment line 9 is correct.

□

### Subclique

Let $c'$ be a subclique of $G^*$ containing *k* alternating elements. We show in this part how to compute the table entries for the sets $\mathcal {C}, \mathcal {F}, \mathcal {A}$ and $\mathcal {E}$. The idea is to construct the table entry by merging successively each table entry of the alternating elements of $c'$. For that, we use at each step an intermediate graph $G_t$ and two intermediate sets $\mathcal {A}_+$ and $\mathcal {E}_+$, defined as follows. Let $L(c') = \{e_1,\dots ,e_k\}$ be a list of alternating elements of $c'$, let $t\le k$, let $E_t=\{e_1,\dots ,e_t\}$, and let $V_t =\bigcup _{e \in E_t} V(G^*(e))$. Let $G_t$ be the subgraph of $G^*$ induced by $V_t$. At step *t*, a solution $S'$ is in $\mathcal {A}_+$ (resp. $\mathcal {E}_+$) if and only if (1) $S'$ is a solution of $G_t$, (2) $S'$ contains a set $C \ne \varnothing$ of closeable paths and (3) $S \setminus C$ is not extensible (resp. extensible).
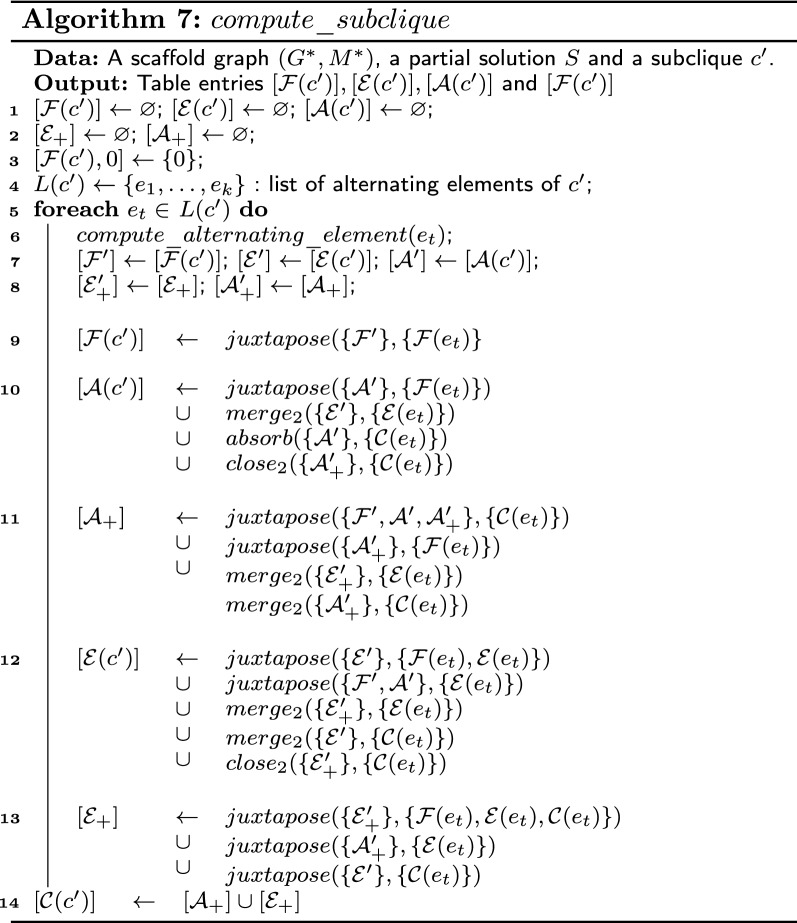


#### Lemma 6

For any subclique $c'$, the value of the table entries provided by Algorithm 7 are correct for the sets $\mathcal {C}(c'), \mathcal {F}(c'), \mathcal {A}(c')$ and $\mathcal {E}(c')$.

#### Proof

Assume table entries returned by $compute\_alternating\_element$ are correct. We show by induction that the values calculated in each step *t* are correct for the graph $G_t$. First, $G_0$ is the empty graph and the unique solution is that containing zero alternating cycles and paths and this solution is frozen. Thus, lines 1 to 3 are correct. Now, consider the alternating element $e_t$ and suppose the previously computed values are correct (*i.e.* values stored in $\mathcal {F}',\mathcal {A}',\mathcal {E}',\mathcal {A}_+'$ and $\mathcal {E}_+'$). Let $S_1$ be a solution in $G_{t-1}$, let $S_2$ be a solution in $G^*(e_t)$ and let $S'$ be a composition of $S_1$ and $S_2$. We have the following properties:

- if $S'$ is obtained by a juxtaposition, then $S_1$ is in $\mathcal {F}',\mathcal {A}',\mathcal {E}',\mathcal {A}_+'$ or $\mathcal {E}_+'$ and $S_2$ is in $\mathcal {C}(e_t),\mathcal {F}(e_t)$ or $\mathcal {E}(e_t)$,
- if $S'$ is obtained by a merge, then $S_1$ is in $\mathcal {E}',\mathcal {A}_+'$ or $\mathcal {E}_+'$ and $S_2$ is in $\mathcal {C}(e_t)$ or $\mathcal {E}(e_t)$,
- if $S'$ is obtained with an absorption, then $S_1$ is in $\mathcal {A}'$ or $\mathcal {A}_+'$ and $S_2$ is in $\mathcal {C}(e_t)$, and
- if $S'$ is obtained by a closing, then $S_1$ is in $\mathcal {A}_+'$ or $\mathcal {E}_+'$ and $S_2$ is in $\mathcal {C}(e_t)$.

Thus, there are 25 complete compositions to consider. If $S_2 \in \mathcal {C}(e_t)$ (resp. $\mathcal {E}(e_t)$) and $S'$ is obtained by a closing (resp. merge), then $S'$ is closeable (resp. extensible) if $S_1$ contains more than one closeable (resp. extensible) alternating path or absorbent, otherwise. Hence, a complete composition obtained with a closing or a merge is not included in a unique set among those defined. This problem can be solved by ignoring certain solutions: $S'$ can be ignored if there is another solution in $G_t$ with the same cardinality.

**Fig. 8:**
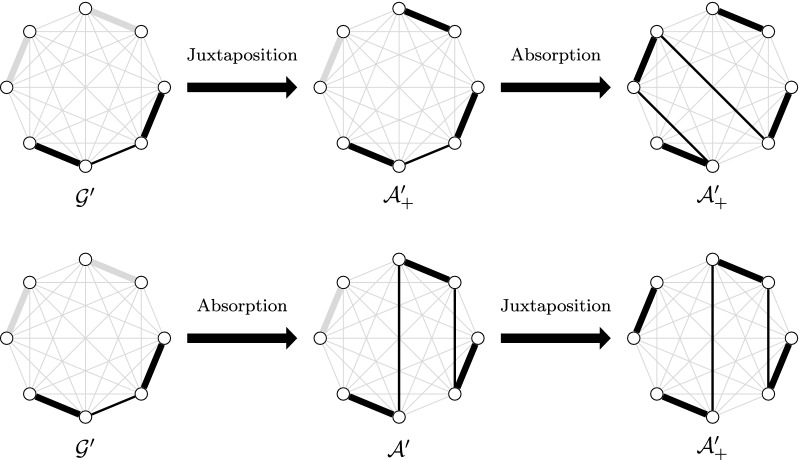
Example of a case ignored by the algorithm. At the top, the solution is obtained after juxtaposing a closeable alternating path $p_1$ and absorbing a closeable alternating path $p_2$. The intermediate solution is in the set $\mathcal {A}'_+$ during the second step. Below, a solution of the same cardinality is obtained after absorbing $p_1$ and juxtaposing $p_2$ and in this case, the intermediate solution is in $\mathcal {A}'$. The upper solution is not considered by the algorithm because the bottom solution has the same cardinality

1. Suppose $S'$ is obtained with a closing (resp. merge) and $S_1$ contains more than one closeable (resp. extensible) alternating path. Let $p_1$ and $p_2$ be closeable (resp. extensible) alternating paths of $S_1$. There is a solution $S'_1$ similar to $S_1$ except that $p_1$ and $p_2$ have been closed into a cycle (resp. merged into a unique alternating path) during a previous step. We can obtain a solution in $G_t$ with the same cardinality as $S'$ by juxtaposing $S'_1$ and $S_2$. Thus, $S'$ can be ignored, and we suppose that a solution obtained with a closing does not contain a closeable alternating path (*i.e.* is not in $\mathcal {A}_+$ or $\mathcal {E}_+$). Likewise, we can suppose a solution obtained with a merge between a solution of $\mathcal {E}'\cup \mathcal {E}_+'$ and a solution of $\mathcal {E}(e_t)$ does not contain an extensible alternating path (*i.e.* is not in $\mathcal {E}(c')$ or $\mathcal {E}_+$).
2. Assume that one of the following conditions is true. (1) $S_1 \in \mathcal {A}_+', S_2 \in \mathcal {E}(e_t)$ and $S'$ is obtained by a merge, (2) $S_2 \in \mathcal {E}_+', S_2 \in \mathcal {F}(e_t)$ and $S'$ is obtained by a merge, (3) $S_1 \in \mathcal {A}_+', S_2 \in \mathcal {F}(e_t)$ and $S'$ is obtained by an absorption. Let *p* be a closeable alternating path of $S_1$ that is absorbed or merged in $S'$. There is a solution $S'_1$ similar to $S_1$ except that all non-matching edges of *p* have been merged or absorbed during previous steps. We can obtain a solution in $G_t$ with the same cardinality as $S'$ by juxtaposing $S'_1$ and $S_2$. Thus, $S'$ can be ignored. Fig. 8 shows an example of case (3).

The second item allows us to ignore three complete compositions: there are 22 still to be considered. Each of these complete compositions is in only one of the six sets of solutions among $\mathcal {F}(c')$, $\mathcal {A}(c')$, $\mathcal {E}(c')$, $\mathcal {A}'_+$ and $\mathcal {E}'_+$.

- Suppose $S'$ is frozen. The only feasible operation to obtain $S'$ is juxtaposition because an addition of an edge of $E(c')\setminus S$ creates an absorbent solution. $S_1$ and $S_2$ are frozen as, otherwise, their juxtaposition is not frozen. Thus, line 9 is correct.
- Suppose $S'$ is absorbent. Thus, $S'$ contains at least one edge in $\mathcal {E}(c')\setminus S$.
  - If $S_2$ is frozen, then the only feasible operation is juxtaposition and $S_1$ is absorbent.
  - If $S_2$ is extensible, then its extensible alternating path is merged with an extensible alternating path of $S_1$ that is not closeable. Thus, $S_2$ is in $\mathcal {E}'$.
  - If $S'$ results from an absorption, then $S_1$ is absorbent and $S_2$ is closeable.
  - If $S'$ results from a closing, then $S_1$ and $S_2$ are closeable. Since the resulting solution is absorbent, $S_1$ is in $\mathcal {A}_+'$.
  Hence, line 10 is correct.
- Suppose $S'\in \mathcal {A}_+$. Then, $S'$ is extensible and does not contain any extensible alternating paths.
  - If $S'$ results from a juxtaposition, then $S_1$ does not contain an extensible alternating path and $S_2$ is either frozen or closeable. In the first case, $S_1$ must be closeable and therefore $S_1\in \mathcal {A}_+'$. In the second case, $S_1$ is in $\mathcal {F}', \mathcal {A}'$ or $\mathcal {A}_+'$.
  - If $S'$ results from a merge, then $S_1$ is closeable and $S_2$ is either extensible or closeable. In the first case, the extensible alternating path of $S_1$ is merged with an extensible alternating path of $S_2$ so that the resulting solution is not extensible. Thus, $S_1$ is in $\mathcal {E}_+'$. In the second case, $S_1$ does not contain an extensible alternating path since otherwise $S'$ is extensible. Thus, $S_1$ is in $\mathcal {A}_+$.
  Hence, line 11 is correct.
- Suppose $S'$ is extensible. Then, either $S_1$ contains an extensible alternating path or $S_2$ is extensible.
  - If $S_1$ is extensible and $S'$ results from a juxtaposition, then $S_2$ is not closeable since otherwise the resulting solution is also closeable. Thus, $S_2$ is frozen or extensible.
  - If $S_1$ is extensible and $S'$ results from a merge. Then, since we only consider solutions of $\mathcal {E}'$ with a unique extensible alternating path, $S_2$ cannot be extensible since otherwise the resulting solution is absorbent. Thus, $S_2$ is closeable.
  - If $S_1$ is in $\mathcal {E}_+'$, then since $S'$ is not closeable, the extensible alternating path of $S_1$ is either merged with an alternating path or closed into a cycle with a closeable alternating path. Thus, $S_2$ is extensible and $S'$ results from a merge or $S_2$ is closeable and $S'$ results from a closing.
  - If $S_2$ is extensible and $S_1$ does not contain any extensible or closeable alternating path, then $S'$ results from a juxtaposition and $S_1$ is frozen or absorbent.
  Hence, line 12 is correct.
- Suppose $S'$ is in $\mathcal {E}_+$. Then, $S'$ is closeable and contains one extensible alternating path. Recall that we ignore solutions resulting from merge between a solution of $\mathcal {E}_+$ and a closeable solution. Thus, $S'$ results from a juxtaposition and either $S_1$ or $S_2$ contains an extensible alternating path.
  - If $S_1$ is in $\mathcal {E}_+'$, then $S_2$ can be any solution.
  - If $S_1$ is in $\mathcal {A}_+'$, then for $S'$ to contain an extensible alternating path, $S_2$ must be extensible.
  - If $S_1$ is extensible, then for $S'$ to contain a closeable alternating path, $S_2$ must be closeable.
  Hence, line 13 is correct.

As after these assignments, each of the solutions of $G_t$ is in a unique set and is a composition of a solution of $G_{t-1}$ and $G^*(e_t)$, computed values for the table entries are correct for $G_t$.

Finally, after the execution of the loop, computed values for sets $\mathcal {F}(c'), \mathcal {A}(c')$ and $\mathcal {E}(c')$ are correct for $G_k = G^*(c')$. It remains to compute the value of the table entry for $\mathcal {C}(c')$. Sets containing closeable alternating paths are exactly the sets $\mathcal {A}_+$ and $\mathcal {E}_+$, thus $\mathcal {A}_+\cup \mathcal {E}_+= \mathcal {C}(c')$. Hence, the assignment line 15 is correct.□

### Clique

Let *c* be a clique of $G^*$ and let *d* be the upper door of *c*. We show in this part how to compute the table entries for the sets $\mathcal {F}(c)$ and $\mathcal {E}(c)$. Note that, since the edge between $G^*(c)$ and its parent is a bridge, the sets $\mathcal {C}(c)$ and $\mathcal {A}(c)$ are empty. Let *e* be the alternating element of *c* containing the upper door *d* of *c*. The idea is to first compute the table entries for the graph $G^*(e)$ and then merge the obtained table entries to the table entries of the subclique. If *e* is an alternating path and *d* is an extremity of *e*, we replace $\mathcal {E}(e)$ by two intermediate sets $\mathcal {\mathcal {E}}_{d}$ and $\mathcal {\mathcal {E}}_{d'}$. Let $S'$ be a solution of $G^*(e)$. Then, $S' \in \mathcal {\mathcal {E}}_{d}$ if and only if $S' \in \mathcal {E}(e)$ and *d* is an extremity of an alternating path of $S'$. Likewise, $S' \in \mathcal {\mathcal {E}}_{d'}$ if and only if $S' \in \mathcal {E}(e)$ and *d* is not an extremity of an alternating path of $S'$. Note that $\mathcal {E}(e)= \mathcal {\mathcal {E}}_{d}\cup \mathcal {\mathcal {E}}_{d'}$. In order to compute these two sets, we reuse the value of $\mathcal {I}_e$, computed in $compute\_alternating\_element$.
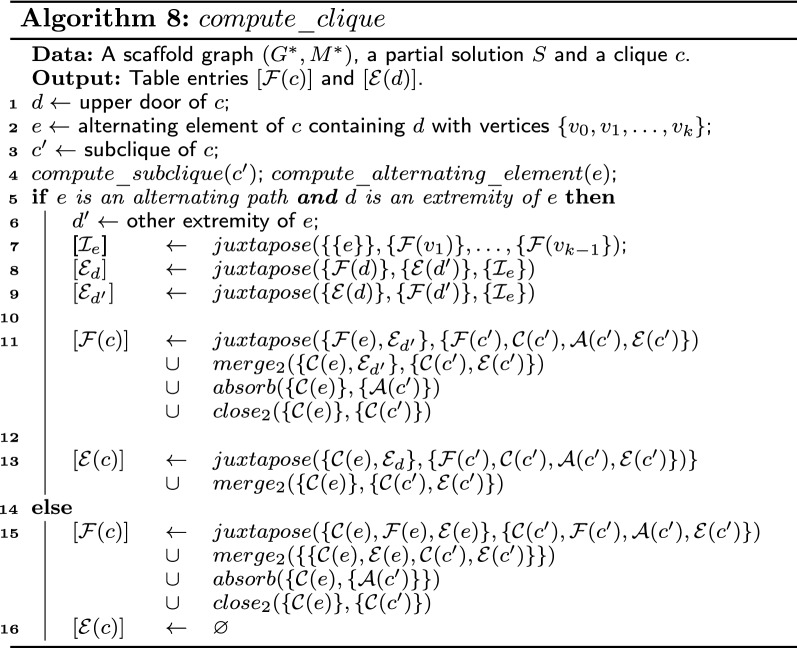


#### Lemma 7

For any clique *c*, the values of the table entries provided by Algorithm 8 are correct for the sets $\mathcal {F}(c)$ and $\mathcal {E}(d)$.

#### Proof

Suppose *e* is an alternating path and the upper door *d* of *c* is an extremity of *e*. Let $d'$ be the other extremity of *e*. First, we compute the table entries for the sets $\mathcal {C}(e),\mathcal {F}(e),\mathcal {\mathcal {E}}_{d}$ and $\mathcal {\mathcal {E}}_{d'}$. Suppose that the values of the table entries provided by $compute\_alternating\_element(p)$ are correct for the sets $\mathcal {C}(e)$ and $\mathcal {F}(e)$. It remains to compute the table entries for the sets $\mathcal {\mathcal {E}}_{d}$ and $\mathcal {\mathcal {E}}_{d'}$. We recall that $\mathcal {I}_e$ is the juxtaposition of all frozen solutions of the inner vertices of *e*.

- A solution $S'$ of $G^*(e)$ is in $\mathcal {\mathcal {E}}_{d}$ if and only if $S'$ is in $\mathcal {E}(e)$ and no non-matching edge is incident to *d* in $G^*(e)$. Thus, $\mathcal {\mathcal {E}}_{d}$ is the juxtaposition of *e*, $\mathcal {I}_e$, $\mathcal {F}(d)$ and $\mathcal {E}(d')$, implying that line 5 is correct.
- Similarly, a solution $S'$ of $G^*(e)$ is in $\mathcal {\mathcal {E}}_{d'}$ if and only if $S'$ is in $\mathcal {E}(e)$ and no non-matching edge is incident to $d'$ in $G^*(e)$. Thus, $\mathcal {\mathcal {E}}_{d}$ is the juxtaposition of *e*, $\mathcal {I}_e$, $\mathcal {E}(d)$ and $\mathcal {F}(d')$, implying that line 6 is correct.

Further, we show that the table entries computed for the set $\mathcal {F}(c)$ and $\mathcal {E}(c)$ are correct.

- A solution $S'$ of $G^*(c)$ is frozen if and only if $S'$ contains an edge incident to *d*. This is the case if the subsolution of $S'$ in $G^*(e)$ is in $\mathcal {F}(e)$ or $\mathcal {\mathcal {E}}_{d'}$ or if *S* is obtained by a merger operation, an absorption operation or a closing operation. Thus, line 8 is correct.
- A solution $S'$ of $G^*(c)$ is extensible if and only if *S* does not contain an edge incident to *d*. This is the case if the subsolution of *S* in $G^*(e)$ is in $\mathcal {C}(e)$ or $\mathcal {\mathcal {E}}_{d}$ or if $S'$ is obtained by a merger operation and $d'$ is an extremity of an alternating path in the subsolution of *S* in $G^*(e)$. Thus, line 10 is correct.

Now, suppose that the upper door *d* of *c* is an inner vertex of *e*. In that case, a subsolution $S'$ of $G^*(c)$ is necessarily frozen. Then any feasible composition of a solution of $G^*(c')$ and a solution of $G^*(e)$ is a frozen solution and thus, line 13 is correct. Similarly, since no extensible solution of $G^*(c)$ exist, line 14 is correct.□

### Feasibility function

We can now provide an answer to the feasibility of finding a solution for $\textsc {Scaffolding}$ by using Algorithm 9. Let *r* be the root of $G^*$. Notice than since *r* does not have an upper door then the subclique of *r* corresponds to *r*. Thus, it is not possible to call $compute\_clique$ on *r*. That is why the first recursive call of the algorithm is made with the function $compute\_subclique$.

#### Corollary 2

Given a partial solution *S*, Algorithm 9 returns true if and only if $(G^*, M^*)$ can be decomposed into $\sigma _p$ alternating paths and $\sigma _c$ alternating cycles. The time complexity of the algorithm is $\mathcal {O}(|V(G^*)| \cdot \sigma _c^2)$.

#### Proof

Since $G^*(root) = G^*$, there is a solution *S* with $\sigma _p(S)=\sigma _p$ and $\sigma _c(S)=\sigma _c$, if and only if *S* is in $\mathcal {C}(root)$, $\mathcal {F}(root)$, $\mathcal {A}(root)$, or $\mathcal {E}(root)$. Thus, the return of the function indicates if such a solution exists and then the algorithm is correct. Concerning the time complexity, the composition operations are executable in $\mathcal {O}(\sigma _c^2)$ time. Thus, without taking into account the recursive calls, the time complexity of Algorithm 5, Algorithm 6, Algorithm 7 and Algorithm 8 in one iteration of a loop is $\mathcal {O}(\sigma _c^2)$. Let *C* denote the number of cliques in *GG*. In Algorithm 5, the number of iterations made by all calls of this function depends on *C* and then the time complexity of all these iterations is $\mathcal {O}(C \cdot \sigma _c^2)$. Similarly, we can show that the time complexities of the iterations made by all calls of Algorithm 6, Algorithm 7 and Algorithm 8 are $\mathcal {O}(|V| \cdot \sigma _c^2)$, $\mathcal {O}(|M^*| \cdot \sigma _c^2)$ and $\mathcal {O}(C \cdot \sigma _c^2)$. Then, the time complexity of all iterations in all functions is $\mathcal {O}((|V)| + |M^*| + C) \cdot \sigma _c^2)$ and since the number of matching edges and the number of cliques is bounded by the number of vertices of $G^*$, we have a time complexity $\mathcal {O}(|V(G^*)| \cdot \sigma _c^2)$.□

**Fig. 9:**
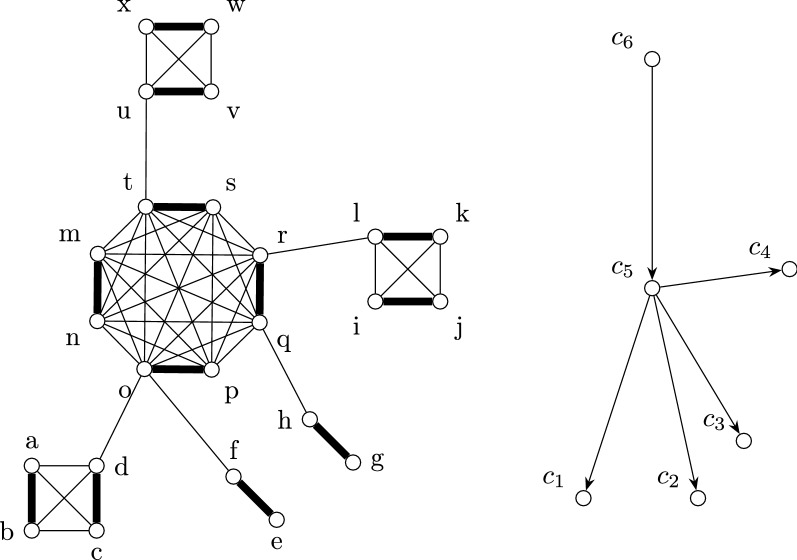
Left: The connected cluster graph $G^*$ used for the pratical example. The graph contains the following cliques: $c_1 =\{a,b,c,d\}$, $c_2 = \{f,e\}$, $c_3=\{h,g\}$, $c_4=\{i,j,k,l\}$, $c_5=\{m,n,o,p,q,r,s,t\}$ and $c_6=\{u,v,w,x\}$. Right: Tree structure of $G^*$ used in the algorithm. The root of these structure is the clique $c_6$

A running example is depicted in Fig. 9 and Example 1 (Tables 1, 2, 3, 4 and 5).
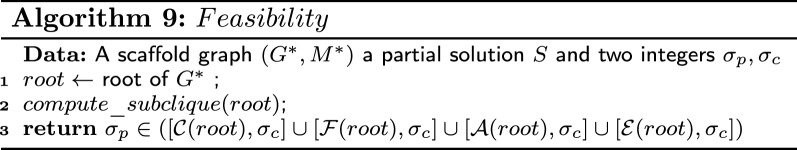

**Table 1:** Compute_vertex

Vertex	#Cycles	$\mathcal {F}$	$\mathcal {E}$
*o*	0	$[2-3]$	$[2-3]$
	1	$\varnothing$	$[1-1]$
	2	$\varnothing$	$\varnothing$
*q*	0	$\varnothing$	$[1-1]$
	$[1-2]$	$\varnothing$	$\varnothing$
*r*	0	$[1-1]$	$[1-2]$
	1	$[0-0]$	$\varnothing$
	2	$\varnothing$	$\varnothing$
*u*	0	$[3-9]$	$[4-10]$
	1	$[2-7]$	$[3-8]$
	2	$[1-5]$	$[2-6]$
Any other vertex	0	$[0-0]$	$\varnothing$
	$[1-2]$	$\varnothing$	$\varnothing$

**Table 2:** Compute_alternating_element

Element	#Cycles	$\mathcal {F}$	$\mathcal {E}$	$\mathcal {C}$
*op*	0	$\varnothing$	$[2-3]$	$[3-4]$
	1	$\varnothing$	$[1-1]$	$[2-2]$
	2	$\varnothing$	$\varnothing$	$\varnothing$
*qr*	0	$[1-2]$	$[2-3]$	$[3-4]$
	1	$\varnothing$	$[1-1]$	$[2-2]$
	2	$\varnothing$	$\varnothing$	$\varnothing$
*uv*	0	$\varnothing$	$[4-10]$	$[4-11]$
	1	$\varnothing$	$[3-8]$	$[3-9]$
	2	$\varnothing$	$[2-6]$	$[2-7]$
Other	0	$\varnothing$	$\varnothing$	$[1-1]$
	$[1-2]$	$\varnothing$	$\varnothing$	$\varnothing$

**Table 3:** Compute_subclique

Subclique	#Cycles	$\mathcal {F}$	$\mathcal {A}$	$\mathcal {E}$	$\mathcal {C}$
$c_1',c_4'$	0	$\varnothing$	$\varnothing$	$\varnothing$	$[1-1]$
	$[1-2]$	$\varnothing$	$\varnothing$	$\varnothing$	$\varnothing$
$c_2',c_3'$	0	$[0-0]$	$\varnothing$	$\varnothing$	$\varnothing$
	$[1-2]$	$\varnothing$	$\varnothing$	$\varnothing$	$\varnothing$
$c_5'$	0	$\varnothing$	$\varnothing$	$[4-6]$	$[4-9]$
	1	$\varnothing$	$[3-7]$	$[3-7]$	$[3-6]$
	2	$\varnothing$	$[2-5]$	$[2-4]$	$[2-5]$
$c_6'$	0	$\varnothing$	$\varnothing$	$[4-10]$	$[4-12]$
	1	$\varnothing$	$[3-10]$	$[3-8]$	$[3-10]$
	2	$\varnothing$	$[2-8]$	$[2-6]$	$[2-8]$

**Table 4:** Compute_clique

Clique	#Cycles	$\mathcal {F}$	$\mathcal {E}$
$c_1,c_4$	0	$[1-1]$	$[1-2]$
	1	$[0-0]$	$\varnothing$
	2	$\varnothing$	$\varnothing$
$c_2,c_3$	0	$\varnothing$	$[1-1]$
	$[1-2]$	$\varnothing$	$\varnothing$
$c_5$	0	$[3-9]$	$[4-10]$
	1	$[2-7]$	$[3-8]$
	2	$[1-5]$	$[2-6]$

**Table 5:** Detailled computation for subclique $c'_5$

Iteration	#cycles	$\mathcal {F}$	$\mathcal {A}$	$\mathcal {A}_+$	$\mathcal {E}$	$\mathcal {E}_+$
$\{m,n\}$	0	$\varnothing$	$\varnothing$	$[1-1]$	$\varnothing$	$\varnothing$
	$[1-2]$	$\varnothing$	$\varnothing$	$\varnothing$	$\varnothing$	$\varnothing$
$\{m,n,o,p\}$	0	$\varnothing$	$\varnothing$	$[3-5]$	$\varnothing$	$[3-4]$
	1	$\varnothing$	$[2-3]$	$[2-3]$	$\varnothing$	$[2-2]$
	2	$\varnothing$	$[1-1]$	$\varnothing$	$\varnothing$	$\varnothing$
$\{e,f,g,h,q,r\}$	0	$\varnothing$	$\varnothing$	$[4-9]$	$[4-6]$	$[4-8]$
	1	$\varnothing$	$[3-7]$	$[3-7]$	$[3-7]$	$[3-6]$
	2	$\varnothing$	$[2-5]$	$[2-5]$	$[2-4]$	$[3-4]$

#### Example 1

Running example on the graph depicted in Fig. 9. Tables 1, 2, 3 and 4 depicte the table entries resulting from Algorithms 5 to 8, respectively. Table 5 display the values of the table entries after each iteration of alternating element for the subclique $c'_5$. Let *c* be the value given by the column “#cycles” and *x* be the item considered in the first column. For each *X* in $\mathcal {F} ,\mathcal {A}, \mathcal {A}_+, \mathcal {E}, \mathcal {E}_+$ and $\mathcal {C}$, the interval given by the column *X* corresponds to [*X*(*x*), *c*].

## Approximation result

We now prove the following approximation result.

### Theorem 4

Algorithm 1 provides a solution for $(\sigma _p,\sigma _c)$-scaffolding in connected cluster graphs with an approximation ratio of at most five and a time complexity $\mathcal {O}(|V|\cdot |E(G^*)| \cdot \sigma _c^2)$. The approximation ratio is tight.

**Proof **We suppose that the input of the algorithm is a scaffold graph $(G^*,M^*,\omega )$ with non-negative weights and such that $G^*$ is a path connected cluster graph. We first show that the algorithm is correct. Note that, since each time we add an edge *e* to *S*, we remove from *E* all incident non matching edges to *e*, the set *S* induces only paths and cycles.

If it is not possible to build a solution from the graph, then the feasibility condition is not verified and then the algorithm returns an error. Otherwise, since we ensure that the feasibility condition is verified at each step, when the algorithm terminates, then it builds $\sigma _p$ paths and $\sigma _c$ cycles.

Now, we prove the approximation ratio. Since they always appear in any solution, we do not consider the edges of $M^*$ in what follows. Notice that, since there is, for each path, one chosen edge less than the number of involved matching edges, and for a cycle, the same number of chosen edge as the number of involved matching edges, then the number of non-matching edges in every solution is exactly $n - \sigma _p$.

We denote by $e_1,\dots ,e_m$ the edges of the graph $G^*$, sorted in non-increasing order by their weights. We denote by $e^A_1,\dots ,e^A_{n-\sigma _p}$ the edges of the solution $S_A$ given by Algorithm 1, sorted in non-increasing order by their weights. In the same way, we denote by $e^{opt}_1,\dots ,e^{opt}_{n-\sigma _p}$ the edges of an optimal solution $S_{opt}$ for the problem, also sorted in non-increasing order. Both sequences $e^A_1,\dots ,e^A_{n-\sigma _p}$ and $e^{opt}_1,\dots ,e^{opt}_{n-\sigma _p}$ are clearly subsequences of $e_1,\dots ,e_m$. Let $\varphi : S_{opt} \rightarrow S_A$ be a mapping such that

1 $$\begin{aligned} \forall e \in S_{opt}, \omega (e) \le \omega (\varphi (e)) \end{aligned}$$

2 $$\begin{aligned} \forall e \in S_A, |\varphi ^{-1}(\{e\})| \le 5 \end{aligned}$$

Inequality (1) indicates that for each $e\in E$ in an optimal solution, there is an edge $\varphi (e) \in S_A$ such that the weight of this latter edge is at least the weight of *e*. Whereas (2) states that for each $e \in S_A$, we may associate *e* to at most four edges of the optimal solution. In the following, we prove that it is possible to define a mapping $\varphi$ satisfying these inequalities.

**Fig. 10:**
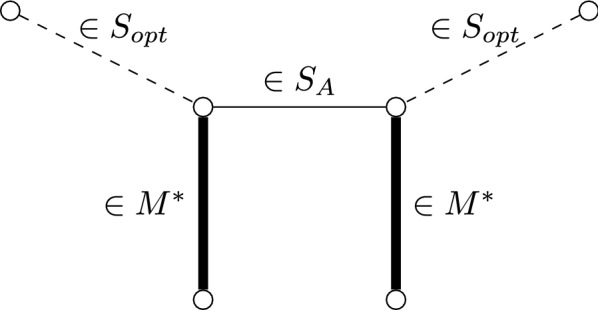
A greedily chosen edge can eliminate up to two optimal edges by the update_edge function

The algorithm may decide not to choose an edge $e^{opt}_i$ for four main reasons:

- $e^{opt}_i$ is eliminated because it is in *R*, when an edge $e^A_j$ is chosen. In this case, we have $\omega (e^A_j) \ge \omega (e_i^{opt})$ because only edges appearing after $e^A_j$ in the ordered list can be in *R*. When an edge $e^A_j$ is chosen, it can eliminate at most two edges of optimal solution by updating of the list of edges (see Fig. 10). We assign $\varphi (e^{opt}_i) = e^A_j$ in this case. (1) is satisfied by construction, and (2) holds when considering only the optimal edges which are eliminated by this way.
- $e^{opt}_i$ is eliminated because its addition disconnects the graph and the number of alternating cycles and alternating paths required to cover the graph becomes too big. This happens in one of the following two cases.
  - $e^{opt}_i$ closes a cycle. In that case, there is at least one edge $e^A_{j}$ in this cycle, and since it has been chosen before the algorithm considers $e^{opt}_i$, we necessarily have $\omega (e^{A}_j) \ge \omega (e^{opt}_i)$. Thus, we assign $\varphi (e^{opt}_i)= e^A_j$. Then, (1) is satisfied by construction. The edge $e^A_j$ has been already chosen, may have eliminated at most two optimal edges, but (2) is still satisfied.
  - $e^{opt}_i$ closes a door *d* and one bridge *dx* incident to *d* is necessary to construct a solution with the remaining edges. There is a door *y* which has been closed by an edge $e^A_j$ in a previous step and this forces *dx* to be in $S_A$. Since closing a door increases by at most one the minimum number of alternating paths required to cover the graph, the closing of *y* forces at most one bridge of $G^*$ to be in $S_A$. Thus, the closing of *y* prevents *d* and *x* from closing, that is, at most two edges of $S^{opt}$, incident to *d* and *x* respectively, can be associated to $e^A_j$ Then, (1) is satisfied by construction. The edge $e^A_j$ may have eliminated at most two optimal edges in *R* and may prevent the closing of a cycle, but (2) is still satisfied.
- $e^{opt}_i$ is eliminated because its inclusion would merge two paths $p_1$ and $p_2$. If $e^{opt}_i$ is not a bridge and $p_1$ and $p_2$ are a single-edge paths, then the number of alternating cycles and paths are reached in *S*, that is $\sigma _c = c$, $\sigma _p = p$ and $S = S_A$. Then, we can find an edge $e^A_j$ such that $|\varphi ^-1(e^A_j)|=0$ and we assign $\varphi (e^{opt}_i) = e^A_j$. Then, (1) and (2) are satisfied by construction. Otherwise, the algorithm eliminates $e^{opt}_i$ because one of the merged paths must be closed into a cycle to reach the correct number of alternating cycles. Otherwise, there is an edge $e^A_j$ in $S_A$ considered before $e^{opt}_i$ in the algorithm such that $|\varphi ^-1(e^A_j)| \le 3$ (since otherwise the path would be already closed into a cycle) and then we assign $\varphi (e^{opt}_i) = e^A_j$. Again, (1) and (2) are satisfied by construction.

From the previous discussion and by (1) and (2), clearly we have:

$$\begin{aligned} \omega (S_{opt}) \le \omega (\varphi (S_{opt})) \le 5 \omega (S_A). \end{aligned}$$

**Fig. 11:**
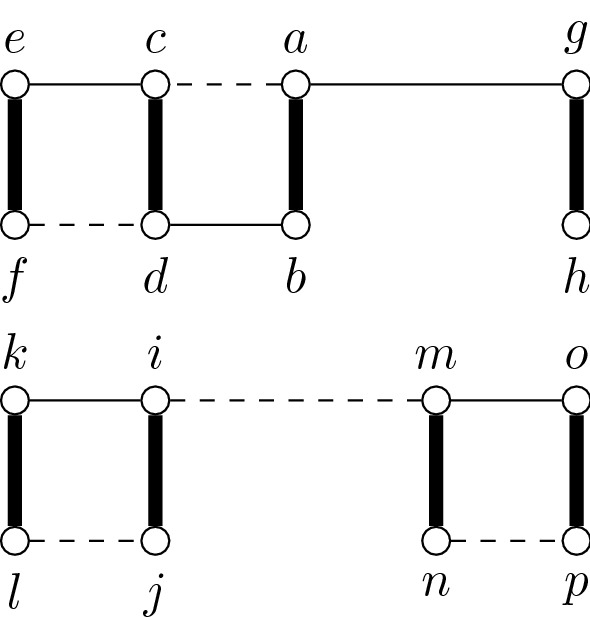
The approximation ratio of five for the greedy algorithm is tight. Matching edges are bold, dashed edges are in the approximate solution and solid edges are in the optimal solution. $G^*$ is composed by the cliques ${C_1 = \{a,b,c,d,e,f\}}$, ${C_2=\{g,h\}}$, ${C_3 = \{i,j,k,l\}}$ and $C_4 = \{m,n,o,p\}$. All edges have weight zero except *ac* and the edges of $S_{opt}$. We suppose that $\sigma _p = 3$ and $\sigma _c=0$, and the greedy algorithm chooses “the wrong edge” *ac* first. Consequently, the solution $S_A$ given by the greedy algorithm is of weight 1, whereas an optimal solution would be of weight 5

The ratio is tight, as shown by the example depicted in Fig. 11.

Concerning the complexity, the edges can be sorted in $\mathcal {O}(|V(G^*)| \log |E(G^*)|)$ time. The feasibility function is called $|E(G^*)|$ times. Thus, the time complexity of the algorithm is $\mathcal {O}(|E(G^*)| \cdot |V(G^*)| \cdot \sigma _c^2)$.

## Experimental results

In this section, we compare the performance of Algorithm 1 with three different feasibility functions and an integer linear programming formulation [15] implemented with ILOG CPLEX [16].

### Dataset

We reuse the dataset already used in [9], which was obtained with the following pipeline:

**Table 6:** Real dataset

Species	Size (bp)	Type	Accession
*Anopheles gambiae* str. PEST (anopheles)	41,963,435	Chromosome 3L	NT_078267.5
*Bacillus anthracis* str. Sterne (anthrax)	5,228,663	Chromosome	NC_005945.1
*Arabidopsis thaliana *(arabido)	119,667,750	Complete genome	TAIR10
*Zaire ebolavirus* (ebola)	18,959	Complete genome	NC_002549.1
*Gloeobacter violaceus* PCC 7421 (gloeobacter)	4,659,019	Chromosome	NC_005125.1
*Lactobacillus acidophilus *NCFM (lactobacillus)	1,993,560	Chromosome	NC_006814.3
*Danaus plexippus* (monarch)	15,314	Mitochondrion	NC_021452.1
*Pandoravirus salinus* (pandora)	2,473,870	Complete genome	NC_022098.1
*Pseudomonas aeruginosa* PAO1 (pseudomonas)	6,264,404	Chromosome	NC_002516.2
*Oryza sativa* Japonica (rice)	134,525	Chloroplast	X15901.1
*Saccharomyces cerevisiae* (sacchr3)	316,613	Chromosome 3	X59720.2
*Saccharomyces cerevisiae* (sacchr12)	1,078,177	Chromosome 12	NC_001144.5

1. Choice of a reference genome, for instance on the nucleotide database from NCBI^2^. Table 6 presents selected genomes used for our experiments. We chose a panel of genomes of various origins and sizes.
2. Simulation of paired-end reads, using wgsim [17]. The chosen parameters are an insert size of 500bp and a read length *L* of 100bp.
3. Assembly using the *de novo* assembly tool, based on a De Bruijn graph efficient representation: minia [18] with *k*-mer size $k=30$.
4. Mapping of reads on contigs, using bwa [19]. This mapping tool was chosen according to results obtained by Hunt [20], a survey on scaffolding tools.
5. Generation of scaffold graph from the mapping file.

Statistics on the numbers of vertices and edges in produced scaffold graphs can be viewed in Table 7.

### Feasibility functions

There is no polynomial-time computable feasibility function in the general case. Thus, to use the greedy algorithm with a specific feasibility function on a real instance, we must transform it. For this, we construct a supergraph by adding edges of weight zero. We compare three feasibility functions, defined on complete graphs, connected cluster graphs and block graphs^3^, respectively. Note that the construction of a complete supergraph requires the largest amount of edge additions whereas the least amount of edge additions is required for the construction of a block supergraph. We already showed in [9] that the computed ratio is close to one on real instances, that is, relatively far from the theoretical ratio of 3. The aim of these experiments is to answer the two following questions:

- Can greedy algorithms on connected cluster graphs and block graphs be used on large scaffold graphs, and what is its associated computation time?
- Do we get a better practical ratio if the amount of additional edges is smaller (e.g. the completion rate, see Table 7, is smaller)? In other words, do we obtain better results on block graphs and connected cluster graphs than in complete graphs?

**Table 7:** Statistics on scaffold graphs

Data	#Contigs	#Edges	$\sigma _p$	Completion rate [%]	
				Cluster	Block
Anopheles	42,045	71,452	7201	27	20
Anthrax	4055	6958	371	94	91
Ebola	17	26	4	81	70
Gloeobacter	4517	7885	506	95	95
Lactobacillus	1898	3335	185	94	89
Monarch	14	19	4	45	39
Pandora	2451	4271	291	91	83
Pseudomonas	5248	9086	543	95	87
Rice	84	26	10	76	69
Sacchr3	296	527	34	88	81
Sacchr12	889	1522	101	94	94

The completion rate is the percentage of added edges compared to number of added edges in the complete version. For all instances, we take $\sigma _c=0$

### Results

Experiments were run on a personal computer with four i7 processors at 1.9GHz and 16GB RAM. Memory usage was very light, even on the biggest instance *anopheles*. Table 8 shows scores and computation times for every instance. We can see that greedy computation times are less than few seconds except for *anopheles*, where the connected cluster graph version and the block graph version need a few minutes. As expected, the greedy algorithms are much faster than the ILP formulation in every case. These results let us answer to our first question: connected cluster graph and block graph versions of the greedy algorithm are capable of treating big instances, however the computation time is significantly bigger than the complete version. Concerning the scores, we can see that the three greedy algorithms have the same score for most of the data. The connected cluster graph and block graph versions have a slightly better score in four instances: *anopheles, anthrax, sacchr3* and *sacchr12*. Moreover, connected cluster graph and block graph versions have the same score in all instances except in *anopheles*, where the block graph version improves the score of the connected cluster graph version by three (which is not really significant compared to the absolute values). These results indicate that the answer to the second question is positive. However, the differences between scores are not significant enough to be completely affirmative. We can think that using the greedy algorithm with feasibility function defined on a sparser class of graphs may lead to better results.

**Table 8:** Results statistics

Data	Complete		Cluster		Block		ILP	
	Score	Time	Score	Time	Score	Time	Score	Time
Anopheles	1,707,529	2.90	1,707,759	99.91	1,707,762	160.77	1,736,748	>3600
Anthrax	226,709	0.26	226,712	0.60	226,712	0.96	228,064	26.22
Abola	776	0.00	776	0.00	776	0.00	776	0.01
Gloeobacter	218,602	0.29	218,602	0.90	218,602	1.38	220,527	14.86
Lactobacillus	95,497	0.12	95,497	0.22	95,497	0.27	96,313	2.48
Monarch	506	0.00	506	0.00	506	0.00	507	0.01
Pandora	119,599	0.16	119,599	0.31	119,599	0.48	120,710	3.85
Pseudomonas	279,607	0.32	279,607	1.18	279,607	1.81	280,978	19.72
Rice	4293	0.00	4293	0.01	4293	0.01	4.320	0.02
Sacchr3	14,524	0.02	14,531	0.03	14,531	0.03	14,623	0.15
Sacchr12	46,041	0.05	46,050	0.07	46,050	0.09	46,395	1.18

The score corresponds to the sum of the weights of the edges. Times are given in seconds

## Conclusion and future work

We presented in this paper the first polynomial-time algorithm approximating the scaffolding problem on non-complete graphs. Using a dynamic programming approach, we exploited the tree-like nature of connected cluster graphs to extend the feasibility function and the analysis of the approximation ratio. We also showed that this new algorithm provides slightly better results on real data than the greedy algorithm on complete graphs, although its theoretical ratio is worse. This leads us to the hypothesis that using a feasibility function defined on a graph class close to the original instance produces better results. This is surprising since, intuitively, algorithms on superclasses can choose from a larger set of edges to build solutions (any solution on the more restricted class is also a solution in the more general class). A natural extension of this work is to consider sparser graphs: for example, one could replace cliques in connected cluster graphs by co-bipartite graphs as the feasibility function is polynomial-time computable in this case [8]. One may also explore the possibility of exploiting randomized algorithms to improve the ratio [6].

## Data Availability

The datasets used and/or analysed during the current study are available from the corresponding author on reasonable request.

## Footnotes

1 Note that *v* follows *u* in the genomic sequence if and only if *u* follows *v* in its reverse complement. Therefore, scaffolds are modeled as undirected graphs in this work.

2 http://www.ncbi.nlm.nih.gov/.

3 A *block graph* is a graph in which every biconnected component is a clique (note that a connected cluster graph is a special case of block graph).
